# Multi-modal proteomic characterization of lysosomal function and proteostasis in progranulin-deficient neurons

**DOI:** 10.1186/s13024-023-00673-w

**Published:** 2023-11-16

**Authors:** Saadia Hasan, Michael S. Fernandopulle, Stewart W. Humble, Ashley M. Frankenfield, Haorong Li, Ryan Prestil, Kory R. Johnson, Brent J. Ryan, Richard Wade-Martins, Michael E. Ward, Ling Hao

**Affiliations:** 1 National Institute of Neurological, Disorders and Stroke (NINDS), National Institutes of Health (NIH), Bethesda, MD USA; 2 Department of Neurodegenerative Disease, UK Dementia Research Institute, Institute of Neurology, University College London, London, UK; 3 Augusta University, University of Georgia Medical Partnership, Athens, GA USA; 4 Cambridge Institute for Medical Research, University of Cambridge, Cambridge, UK; 5 Medical Scientist Training Program, Feinberg School of Medicine, Northwestern University, Chicago, IL USA; 6 Department of Physiology, Anatomy and Genetics, Oxford Parkinson’s Disease Centre, Kavli Institute for Nanoscience Discovery, University of Oxford, Dorothy Crowfoot Hodgkin Building, South Parks Road, Oxford, OX1 3QU UK; 7 Department of Chemistry, George Washington University, Washington, DC USA

**Keywords:** Lysosome, Neuron, Progranulin, Proteomics, Half-life, Turnover, Frontotemporal dementia, iPSC, PGRN, dSILAC

## Abstract

**Background:**

Progranulin (PGRN) is a lysosomal glycoprotein implicated in various neurodegenerative diseases, including frontotemporal dementia and neuronal ceroid lipofuscinosis. Over 70 mutations discovered in the *GRN* gene all result in reduced expression of the PGRN protein. Genetic and functional studies point toward a regulatory role for PGRN in lysosome functions. However, the detailed molecular function of PGRN within lysosomes and the impact of PGRN deficiency on lysosomes remain unclear.

**Methods:**

We developed multifaceted proteomic techniques to characterize the dynamic lysosomal biology in living human neurons and fixed mouse brain tissues. Using lysosome proximity labeling and immuno-purification of intact lysosomes, we characterized lysosome compositions and interactome in both human induced pluripotent stem cell (iPSC)-derived glutamatergic neurons (i^3^Neurons) and mouse brains. Using dynamic stable isotope labeling by amino acids in cell culture (dSILAC) proteomics, we measured global protein half-lives in human i^3^Neurons for the first time.

**Results:**

Leveraging the multi-modal proteomics and live-cell imaging techniques, we comprehensively characterized how PGRN deficiency changes the molecular and functional landscape of neuronal lysosomes. We found that PGRN loss impairs the lysosome’s degradative capacity with increased levels of v-ATPase subunits on the lysosome membrane, increased hydrolases within the lysosome, altered protein regulations related to lysosomal transport, and elevated lysosomal pH. Consistent with impairments in lysosomal function, *GRN*-null i^3^Neurons and frontotemporal dementia patient-derived i^3^Neurons carrying *GRN* mutation showed pronounced alterations in protein turnover, such as cathepsins and proteins related to supramolecular polymerization and inherited neurodegenerative diseases.

**Conclusion:**

This study suggested PGRN as a critical regulator of lysosomal pH and degradative capacity, which influences global proteostasis in neurons. Beyond the study of progranulin deficiency, these newly developed proteomic methods in neurons and brain tissues provided useful tools and data resources for the field to study the highly dynamic neuronal lysosome biology.

**Graphical Abstract:**

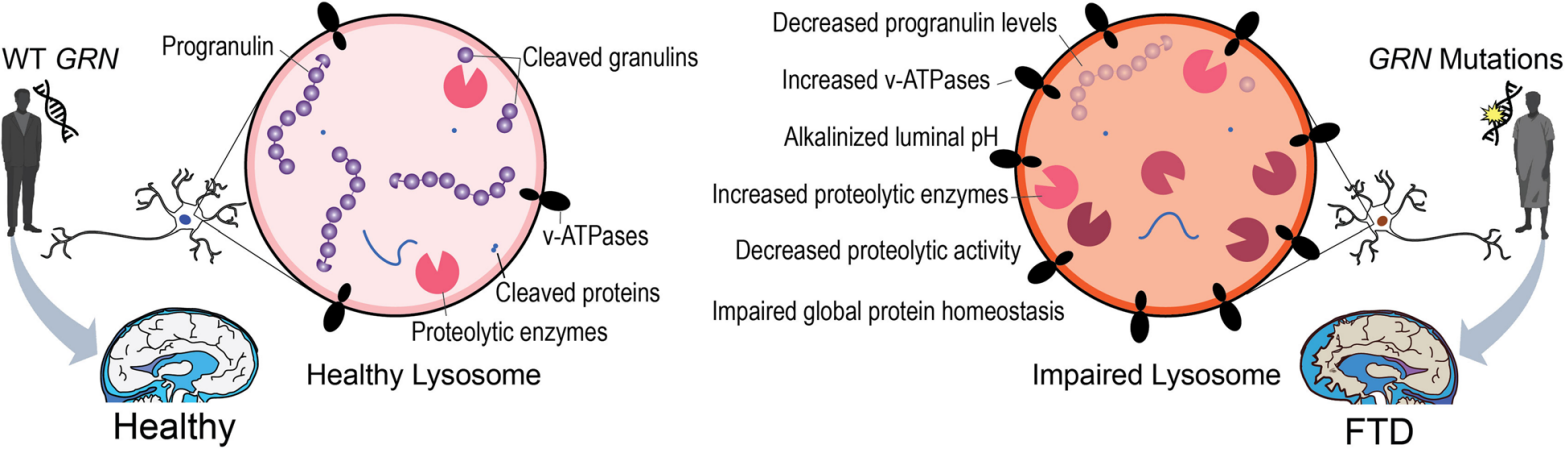

**Supplementary Information:**

The online version contains supplementary material available at 10.1186/s13024-023-00673-w.

## Background

As the primary degradative organelle of the cell, lysosome orchestrates proteostasis via the autophagy-lysosome pathway and degrades macromolecules such as proteins, lipids, carbohydrates, and RNA [1–3]. Neurons are particularly sensitive to lysosomal perturbations, as evidenced by numerous neurodegeneration-related mutations in genes that regulate lysosomal biology [4–6]. In particular, pathogenic mutations in genes that encode lysosomal or lysosome-associated proteins (e.g., *GRN, LRRK2, GBA, TMEM106B, C9orf72*) are major causes of inherited neurodegenerative diseases [5–7]. Genetic mutations associated with defective lysosomal enzymes lead to the accumulation of degradative substrates within the lysosomal lumen, consistent with chronic lysosomal dysfunction [8]. However, the molecular mechanisms by which many of these mutated genes cause lysosomal dysfunction and disease remain unclear.

Mutations in the *GRN* gene cause inherited frontotemporal dementia (FTD) and have also been linked to other neurodegenerative diseases, including neuronal ceroid lipofuscinosis (NCL), Alzheimer’s disease (AD) and Parkinson’s disease (PD) [9–12]. Over 70 pathogenic mutations in the *GRN* gene have been discovered, and all of these mutations result in reduced expression of the progranulin (PGRN) protein [13–15]. Progranulin is trafficked to the lysosome and cleaved by cathepsins into smaller intra-lysosomal peptides called granulins [16]. Functionally, progranulin loss leads to a host of lysosome-related phenotypes, including defective autophagy and autophagosome-lysosome fusion [17, 18]. Recently, lysosomal lipid dysregulation was found to be a major element of *GRN*-related disease pathogenesis [19, 20]. However, the molecular cascade by which loss of intra-lysosomal progranulin impacts lysosomal biology and eventually leads to FTD remains elusive.

Capturing the dynamic lysosomal activities in highly polarized neurons is a challenging task, particularly in a high-throughput fashion. Our human induced pluripotent stem cells (iPSCs)-derived glutamatergic neuron (i^3^Neuron) [21–23] platform provides pure and scalable human neurons and can be genetically edited to create *GRN*-null neurons as a neuronal model to study progranulin deficiency. Recent advances in capturing organelle dynamics have provided useful tools, such as proximity labeling in living cells via engineered ascorbate peroxidase (APEX) [24] or biotin ligases [25], immunopurification of intact organelles [26], and biotinylation by antibody recognition (BAR) [27] in primary tissues, though mostly in non-neuronal contexts. Other proteomics-based studies in progranulin mouse models mostly captured global changes regardless of cell type or organelle [28–30]. Developing proteomic techniques to probe lysosomes in neurons can provide valuable insights into the converging pathways of lysosomal dysfunctions in neurodegenerative diseases. We recently developed a lysosome proximity labeling method (Lyso-APEX) to characterize the dynamic lysosome interactome in i^3^Neurons [31–33]. In this study, we further expanded the lysosome toolbox by implementing the immunopurification of intact lysosomes (Lyso-IP) technique in our i^3^Neuron platform and Lyso-BAR technique in fixed mouse brain tissues. We comprehensively characterized lysosomal content and interactions using Lyso-APEX and Lyso-IP in i^3^Neurons and Lyso-BAR in fixed mouse brain tissues. To characterize global proteostasis in human neurons, we also designed a dynamic stable isotope labeling by amino acids in cell culture (dSILAC) [34] proteomic method that was suitable for iPSC-derived neuron cell type to measure global protein half-lives in i^3^Neurons for the first time.

Leveraging these multifaceted proteomic techniques, we systematically characterized the impact of progranulin loss using multi-modal readouts of lysosomal biology in i^3^Neurons and mouse brains. We found that loss of PGRN in human neurons presented increased levels of v-ATPase subunits on the lysosome membrane, increased catabolic enzymes within the lysosome, and elevated lysosomal pH. Mouse brains lacking PGRN also present elevated levels of lysosomal catabolic enzymes and bi-directional protein changes related to lysosomal transport. Using fluorescence microscopy, we confirmed that PGRN-deficient lysosomes are less acidic and have decreased cathepsin B enzymatic activity compared to WT lysosomes. Consistent with impairments in protein homeostasis, *GRN*-null i^3^Neurons have pronounced alterations in protein turnover, which was validated by FTD patient-derived i^3^Neurons carrying *GRN* mutation. Collectively, these results show that progranulin loss leads to a downstream molecular cascade involving lysosomal alkalinization and decreased degradative capacity, thereby impacting neuronal proteostasis. Multiple downstream proteins affected by these changes are involved in neurodegenerative pathways, suggesting molecular convergence of multiple neurodegeneration-related genes at the lysosome.

## Methods

### Human i^3^Neuron culture

Human iPSC-derived cortical neurons (i^3^Neurons) were cultured based on our previously established protocol [23]. Briefly, human iPSCs were maintained on Matrigel (Corning Incorporated # 354277) coated tissue culture dishes in Essential 8 medium (Gibco #A1517001). Five iPSC lines were used in this study, as listed in Table 1. Four to six biological replicates were used for each i^3^Neuron cell line per comparison group. A doxycycline-inducible neurogenin2 (NGN2) cassette (Addgene # 105840) was stably integrated into each iPSC line, enabling rapid differentiation to glutamatergic cortical neurons (i^3^Neurons) in a week. Between day 0 and day 3, iPSCs were maintained in neuronal induction medium [23]. Day-3 neurons were replated on poly-L-ornithine coated plates in Brainphys neuron medium and maintained by half-medium change every two days until neuronal maturation in two weeks.

**Table 1:** List of human iPSC lines used in this study

Cell line	Description	Source
WT	WTC 11 line, Healthy 30-year-old Japanese male donor	Coriell Institute #GM25256
*GRN* KO	WTC11 line with a 7 base pair insertion in one GRN allele and 10 base pair deletion in the other *GRN* allele resulting in complete loss of function	Generated in house
ptMut	FTD patient cell line harboring a heterozygous *GRN* mutation (c.26 C > A, p.A9D)	Dr. Dimitri Krainc [35]
ptWT	Isogenic control line by correcting the *GRN* mutation in ptMut line	Dr. Dimitri Krainc [35]
ptKO	Complete knock out of *GRN* in ptWT line using CRISPR-Cas9	Generated in house

### Animals

WT (C57BL/6 J) and *GRN*^*−/−*^ (B6.129S4(FVB)-Grntm1.1Far/Mmjax, MMRRC stock#036771-JAX) mice were obtained from the Jackson Laboratory and housed in the NIH animal facility [36]. Whole brains were dissected from 20-month-old male WT and *GRN*^*−/−*^ mice after cardiac perfusion with 4% paraformaldehyde (PFA). Cortex was fixed in 4% PFA overnight, incubated in 30% sucrose for 24 h, and snap-frozen on dry ice. A microtome was used to generate 40 μm thick coronal slices that were stored in cryoprotectant at -30°C.

### Lysosomal proximity labeling in i^3^Neurons

Lysosomal proximity labeling was achieved by stable integration of ascorbate peroxidase (APEX2) enzyme onto the C terminus of LAMP1 protein in human iPSCs and differentiating iPSCs into i^3^Neurons, as the previously established KuD-LAMP1-APEX (Lyso-APEX) line [32]. A cytosolic localized nuclear exporting signal (NES) APEX i^3^Neuron line was used as the spatial control [31, 32]. Prior to proximity labeling, i^3^Neurons were incubated in 500 μM biotin-tyramide (Adipogen, # 41994–02-9) for 30 min in a CO_2_ incubator. Proximity labeling was induced by incubating the cells in 1 mM of hydrogen peroxide for exactly 1 min followed by rapid quenching using ice-cold quench buffer (10 mM sodium azide, 10 mM sodium ascorbate, 5 mM TROLOX in PBS). Neurons were lysed with cold lysis buffer (50 mM Tris–Cl pH 7.4, 500 mM NaCl, 0.2% SDS, 1 mM DTT, 10 mM sodium azide, 10 mM sodium ascorbate, 5 mM TROLOX, cOmplete mini protease inhibitor tablets). Detailed sample preparation procedures have been described previously [33]. Briefly, neuron lysates were sonicated with QSonica (Q800R) sonicator for 15 min at 2 °C and clarified by centrifugation. Total protein concentrations were measured using a detergent-compatible (DC) Colorimetric Protein Assay (Bio-Rad # 5000111). Biotinylated proteins were enriched with streptavidin (SA) magnetic beads (Cytiva, # 28–9857-99) for 18 h rotating at 4 °C and washed extensively to reduce nonspecific bindings. Biotinylated proteins were reduced, alkalized, and digested into peptides on the SA beads. The optimal SA beads-to-protein ratio and trypsin-to-SA beads ratio were previously determined [32]. After overnight digestion using Trypsin/Lys-C (Promega, #V5073), supernatant was collected from the magnetic beads, and the digestion reaction was quenched with 10% trifluoroacetic acid until pH < 3. Peptides were desalted with a Waters Oasis HLB 96-well extraction plate, dried under SpeedVac, and stored at -30 °C until LC–MS analysis.

### Rapid lysosome immunopurification from i^3^Neurons

Lysosome Immunopurification (Lyso-IP) iPSC line was generated by the stable expression of LAMP1-3xHA in WT and *GRN* KO iPSC lines. i^3^Neurons were differentiated as described above and maintained in 15 cm dishes until day 14. A control i^3^Neurons line without HA expression (mEmerald) was used to control nonspecific labeling background. Neurons were washed 2 times with PBS and dissociated from the plate using forceful pipetting of 10 ml of PBS. Next, neurons were resuspended in 1 ml cold KPBS (136 mM KCl, 10 mM KH2PO4, pH 7.25 adjusted with KOH) and gently homogenized with 21 strokes through an isobiotec balch-style cell homogenizer with a 10 μm ball bearing. Each neuron lysate sample was incubated with 150 μL of pre-washed anti-HA magnetic beads (Thermo # 88836/88837) for 3 min on a rotator and gently washed three times with KPBS. Beads bound with intact lysosomes were resuspended in 100 μl of Lyso-IP lysis buffer (50 mM HEPES, 50 mM NaCl, 5 mM EDTA, 1% SDS, 1% TritonX, 1% NP-40, 1% Tween 20, 1% deoxycholate, 1% glycerol, 5 mM TCEP) and heated at 60 °C for 30 min at 1000 g agitation. The supernatant was collected, and the beads were washed with an additional 50 μl of lysis buffer. Supernatant was combined into a new tube for routine bottom-up proteomics steps as described below.

### Lysosomal proximity labeling in mouse brains

Mouse brain slices were picked evenly throughout the whole brain and washed with PBS three times. Endogenous peroxidase activity in brain slices was quenched with 0.3% H_2_O_2_ in PBS for 30 min. The slices were blocked using 3% donkey serum and 0.25% TritonX in PBS followed by primary antibodies in blocking buffer at 4 °C on a rocker overnight. After the slices were washed thoroughly with PBST, they were incubated with secondary antibody conjugated to HRP in blocking buffer for 1 h at room temperature and extensively washed in PBST. The slices were then incubated in biotin-tyramide with 1% fetal bovine serum (FBS) in PBS for 30 min, and then 0.003% H_2_O_2_ for 10 min, immediately followed by washing with quench buffer (10 mM sodium azide and 500 mM sodium ascorbate). Brain slices without primary antibody treatment were used as the negative control group to compare with Lyso-BAR. One slice from each group was further treated with appropriate Alexa Fluor for microscopy imaging. Twenty brain slices from each group were transferred to 100 μL of Lyso-BAR lysis buffer (3% SDS + 2% sodium deoxycholate in PBS), boiled at 99°C for 1 h at 1200 g agitation, and sonicated with QSonica Sonicator for 15 min. The lysate was boiled again at 99 °C for an additional 30 min until all tissues were homogenized and dissolved into solution. The lysate was diluted using PBS to reduce SDS concentration and clarified by centrifugation. Biotinylated proteins were enriched following the same steps described above for Lyso-APEX sample preparation with optimized SA beads-to-protein ratio and trypsin-to-beads ratio for Lyso-BAR samples.

### Dynamic SILAC proteomics in i^3^Neurons

Human i^3^Neurons were maintained on PLO coated 12-well dishes in light amino acid-containing media (DMEM:F12 for SILAC medium (Athena Enzyme Systems #0423), N2 Supplement (Life Technologies Corporation # 17502048), B27 Supplement (Life Technologies #NC1001496), NEAA (Life Technologies # 11140050), GlutaMAX (Life Technologies # 35050061), BDNF (PeproTech #450–02), NT-3 (PeproTech #AF-450–03-100ug), 0.3 mM of Arginine (Sigma #A4599), and 0.5 mM of Lysine (Sigma #L7039)). On day 10 of i^3^Neuron culture, neurons were gently washed with PBS twice and switched into media containing the same components except for replacing light lysine with heavy stable isotope-labeled (^13^C_6_^15^N_2_) lysine (Cambridge Isotope Laboratories #CNLM-291-H-PK). For multiple time point experiments, neurons were harvested at 1, 2, 4, and 6 days (accurate to within 10 min) after media switch. For single time point experiments, neurons were harvested after 4 days (96 h) of media switch. Neurons were gently washed with PBS twice, lysed in 100 µL of ice-cold lysis buffer containing 0.1% Rapigest (Waters # 186008740), 150 mM NaCl, and 50 mM Tris–HCl, sonicated for 15 min, and clarified by centrifugation. Total protein concentrations were determined by DC Protein assay (BioRad). Protein disulfide bonds were reduced by 5 mM of Tris(2-carboxyethyl) phosphine (TCEP) for 30 min, followed by addition of 15 mM of iodoacetamide (IAA) for 30 min in a ThermoMixer shaking at 800 g at 37 °C. Proteins were digested with LysC (Promega #VA1170) at 1:30 (enzyme: protein) ratio for 16 h at 37 °C and quenched with 10% trifluoroacetic acid (TFA) until pH < 3. Peptides were desalted using a Waters Oasis HLB 96-well extraction plate based on the manufacturer’s protocol. Peptide samples were dried under SpeedVac and stored at -80 °C until LC–MS analysis.

### LC–MS/MS analysis

LC–MS/MS analyses were conducted on a Dionex UltiMate3000 nanoLC system coupled with a Thermo Scientific Q-Exactive HFX or a Fusion Lumos mass spectrometer. Before injection, peptide samples were reconstituted in 2% acetonitrile (ACN), 0.1% formic acid (FA) in LC–MS grade water and centrifuged to collect supernatant. Easy-spray PepMap C18 columns (2 µm, 100 Å, 75 µm × 75 cm) were used for peptide separation with a flow rate of 0.2 µL/min and column temperature of 60 °C. The mobile phase buffer A was 0.1% FA in water, and buffer B was 0.1% FA in acetonitrile. A two-hour gradient was used for proximity labeling proteomics, and a three-hour gradient was used for SILAC proteomics. LC–MS/MS analyses were conducted with a top 40 data dependent acquisition with MS range of m/z 400–1500, MS resolution of 120 K, isolation window of m/z 1.4, dynamic exclusion of 22.5 s, and collision energy of 30% for higher-energy collisional dissociation (HCD) fragmentation. Automatic gain control (AGC) targets were 1 × 10^6^ for MS and 2 × 10^5^ for MS/MS. Maximum injection times (maxIT) were 30 ms for MS and 35 ms for MS/MS. Targeted proteomics was conducted in the parallel reaction monitoring (PRM) mode using an inclusion list generated based on untargeted DDA data with an isolation window of m/z 1.4, retention time window of 5 min, and maxIT of 100 ms.

### Proteomics data analysis

LC–MS/MS raw files from Lyso-APEX, Lyso-IP, and Lyso-BAR proteomic experiments were analyzed with Thermo Fisher Proteome Discoverer (2.4.1.15) software. For dynamic SILAC proteomic data, MaxQuant (1.6.17.0) software was used for data analysis. Targeted PRM proteomics data was analyzed using the Skyline [37] software. High abundant unique peptides were selected for each target protein based on untargeted DDA data. Swiss-Prot *Homo sapiens* database was used for i^3^Neuron data and *Mus musculus* database was used for mouse data with 1% false discovery rate (FDR) for protein identification. Custom-made contaminant protein libraries (https://github.com/HaoGroup-ProtContLib) were included in the data analysis pipeline to identify and remove contaminant proteins [38]. Trypsin was selected as the enzyme with a maximum of two missed cleavages. Cysteine carbamidomethylation was included as fixed modification, and oxidation of methionine and acetylation of the protein N-terminus were selected as variable modifications.

Protein/peptide identification and peak intensities were output as excel files for further analysis using Python or R. Statistical analyses (t-test) and volcano plots for Lyso-APEX, Lyso-IP, and Lyso-BAR proteomics were conducted in Python. Lyso-APEX and Lyso-BAR data were normalized to the most abundant endogenously biotinylated protein (PCCA) before statistical analysis as described previously [32]. For dynamic SILAC data, Maxquant output files were further processed with R to calculate heavy/light peptide ratios and construct the degradation and synthesis curves as well as curve-fitting to the first-order kinetic in multiple time point experiment. For single time point experiments, peptide level Maxquant output files were processed with Python to calculate the peptide half-lives using the equation: t_1/2_ = t_s_ × [ln2 / ln (1 + Ψ)], where t_s_ represents the sampling time after media switch, and Ψ represents the heavy-to-light abundance ratio of the peptide. The recycling of intrinsic amino acids from protein degradation was not considered here since amino acid recycling in cell culture is not as problematic as in the whole animal [39]. Apparent protein level half-lives were calculated by averaging the half-lives of unique peptides belonging to the specific protein. Statistical analysis was conducted with t-test and *p*-values were corrected for multiple hypothesis testing with Benjamini–Hochberg procedure in R. Further data merging, normalization, and filtering were conducted in Python. Protein GO enrichment analysis was conducted using ShinyGO [40]. Protein network analysis was conducted with STRING [41].

### Live cell ratiometric pH assay

Live cell ratiometric lysosomal pH measurements were conducted using a modified method from Saric et al [42], further optimized for high-content imaging and analysis. WT and *GRN*-KO i^3^Neurons were maintained on 96-well dishes. On day 10, neurons were loaded with 50 μg/mL pH-sensitive Oregon Green-488 dextran (Invitrogen, #D7171), and 50 μg/mL pH-insensitive/loading control Alexa Fluor-555 red dextran (Invitrogen, #D34679) for 4 h, before washing three times with PBS then chased overnight with neuronal media after PBS washes the day before imaging. These dextrans accumulate in lysosomes, and high-content microscopy quantification of their fluorescence enables ratiometric calculations of pH within individual lysosomes. Physiological buffers of known pH (4–8) containing 10 μg/mL nigericin were placed on WT neurons to generate a calibration curve. Live cell spinning disk confocal microscopy was performed using an Opera Phenix HCS System (PerkinElmer); calibration and sample wells were imaged at 63 × ; counterstaining was done with NucBlue Live ReadyProbes Reagent (Invitrogen, #R37605) to count and segment nuclei. Lysosome pH was calculated as ratiometric measurement of lysosomes (488/555 nm), with subsequent calculation of the pH of those compartments based on the corresponding calibration curve. All analysis was performed using PerkinElmer’s Harmony HCA Software (PerkinElmer). Statistical analyses for all imaging data were conducted using an independent student’s t-test.

### Magic red cathepsin B activity assay

Human i^3^Neurons were plated at a density of 50,000 cells on PLO-coated ibidi slides (Ibidi # 80827) and maintained to day 10. Magic Red (Abcam #AB270772-25TEST) was added to the cells at 1:25 final dilution and incubated in the dark for 30 min at 37 °C. Cells were washed twice with PBS and incubated with Hoechst 33342 (Thermo Scientific # 62249) at 1:10,000 for 5–10 min and then washed with PBS. Neurons were imaged using Nikon spinning disk confocal at 60 × oil objective. Images were edited and analyzed using ImageJ software [43]. Statistical analysis was conducted using an independent student’s t-test.

### Live cell DQ-BSA Assay in i^3^Neurons

WT and *GRN*-KO i^3^Neurons were plated on 384-well dishes. On day 10, neurons were incubated with 45 μg/mL DQ-BSA Red (Invitrogen, #D12051) for 5 h to allow for substrate endocytosis. Live cell spinning disk confocal microscopy was performed via Opera Phenix HCS System (PerkinElmer); control and sample wells were imaged at 40 × and counterstaining was done with NucBlue Live ReadyProbes Reagent (Invitrogen, #R37605) to count and segment nuclei. All analysis was performed via PerkinElmer’s Harmony HCA Software (PerkinElmer).

### Western blotting

Intact lysosomes were isolated via immunopurification as described above. The intact lysosomes on beads were boiled with sample buffer at 95°C for 5 min. The beads were magnetized, and the supernatant was used for immunoblotting. Lysates were separated using 4–15% precast polyacrylamide gels (Bio-Rad, # 4561083) at 100 V and then transferred using the Trans-Blot Turbo transfer kit onto nitrocellulose membranes (Bio-Rad, # 1704270). Membranes were blocked with 5% nonfat dry milk prepared in TBST (Tris-buffered saline with Tween 20) for 1 h at room temperature and probed with primary antibodies in 5% bovine serum albumin (BSA) in TBST at 4°C overnight (See Supplementary Table S1 for antibodies and dilutions). Following incubation, membranes were washed 3 × with TBST and incubated in secondary antibodies diluted 1:5000 in 5% BSA for 1 h at room temperature. Membranes were then washed 3 × with TBST and visualized using ECL western blotting substrate.

### Fluorescence imaging

i^3^Neurons were cultured on PLO-coated ibidi slides (Ibidi # 81,506) for fluorescence imaging. Neurons were fixed in 4% PFA for 10 min, washed very gently with PBS, and incubated in blocking buffer (1% bovine serum albumin + 0.1% TritonX) for 1 h at room temperature (RT). Next, neurons were incubated with primary antibody in blocking buffer overnight at 4 °C, gently washed with PBS, and incubated in secondary antibody for 1 h at RT. Following thorough washes, neurons were ready to be imaged. Mouse brain slices were prepared in the same steps as neuron culture for fluorescence imaging. All antibodies and their respective applications and dilutions are listed in Supplementary Table S1. Confocal images were obtained using a Nikon Eclipse Ti spinning disk confocal microscope at 60 × using an oil immersion objective with constant setting between experimental groups. Data analysis was conducted in ImageJ.

## Results

### Multi-modal proteomics captures holistic lysosomal biology

**Fig. 1:**
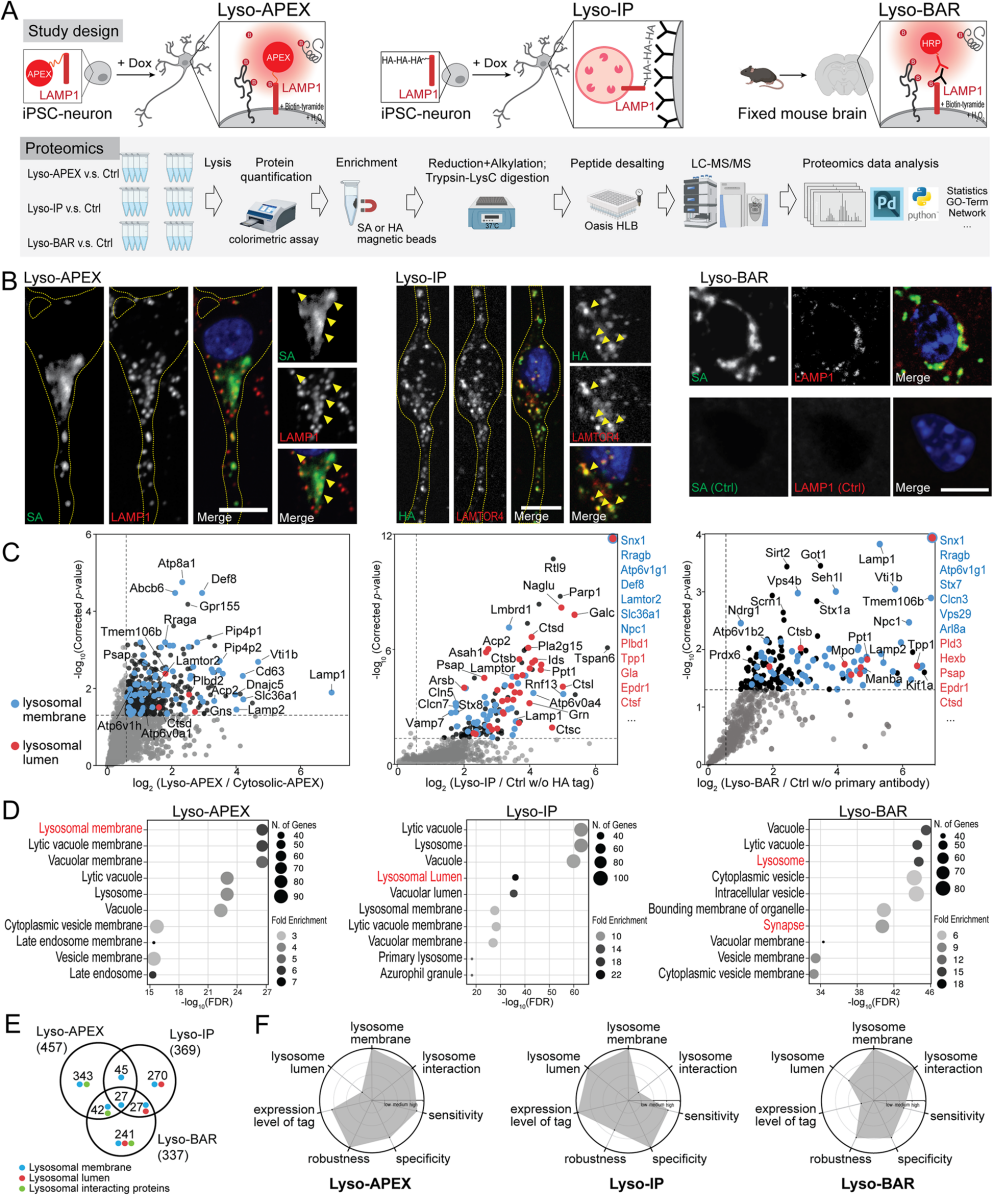
A map of the lysosomal proteome and interactome in human neurons and mouse brains. **A** Schematics of lysosomal proximity labeling (Lyso-APEX) in i^3^Neurons, lysosomal immunopurification (Lyso-IP) in i^3^Neurons, and lysosomal biotinylation by antibody recognition (Lyso-BAR) in fixed mouse brain tissues. **B** Fluorescence imaging of Lyso-APEX, Lyso-IP, and Lyso-BAR activities in i^3^Neurons and fixed mouse brains. Biotinylated proteins, stained with streptavidin (SA-488), colocalize with lysosomal markers in i^3^Neurons and fixed mouse brain tissues. HA-tagged lysosomes colocalize with lysosomal markers in i^3^Neurons. Nuclei were stained by Hoechst. Scale bars are 10 μm. **C** Volcano plots showing significantly enriched proteins from WT Lyso-APEX compared to cytosolic-APEX, Lyso-IP compared to control neurons without HA-LAMP1 expression, and Lyso-BAR compared to control mouse brains without LAMP1 primary antibody staining (*N* = 4 for each group). Dotted lines denote corrected *p*-value of 0.05 (y-axis) and ratio of 1.5 (x-axis). The known lysosomal membrane and lumen proteins are highlighted in blue and orange colors, respectively. **D** GO enrichment analyses of significantly enriched proteins in Lyso-APEX, Lyso-IP, and Lyso-BAR proteomics. **E** Venn diagram comparison of significantly enriched proteins in three proteomics methods. **F** Radar plot comparison of three methods regarding the proteome coverage (lysosome lumen, membrane, and interaction), tag expression level, sensitivity, specificity, and robustness

Lysosomes play critical roles in neurons, such as degradation, endocytosis, signal transduction, nutrient sensing, and long-distance trafficking through axons [44–46]. Different methods of characterizing lysosomal composition and interactions now exist, each with its own strengths [33, 47, 48]. However, a comprehensive characterization of lysosomal biology in neurons with these modern tools has not been performed. Here, we developed three complementary proteomic strategies to characterize lysosomal contents and dynamic lysosomal interactions in both living human neurons and fixed mouse brain tissues (Fig. 1A). Lysosome proximity labeling using ascorbate peroxidase (Lyso-APEX) captured lysosome membrane proteins and lysosome interactions in i^3^Neurons. Rapid lysosomal immunopurification (Lyso-IP) provided both lysosome lumen and membrane proteins from i^3^Neuron. Lysosomal biotinylation by antibody recognition (Lyso-BAR) revealed lysosome proteins and interactions in situ from fixed mouse brain tissues. The proper locations of these probes were validated by immunofluorescence and western blotting (Fig. 1B and Supplementary Figure S1). Control groups were carefully selected for each probe to reduce nonspecific labeling and ensure intracellular spatial specificity (Fig. 1C).

Lyso-APEX, Lyso-IP, and Lyso-BAR proteomics provided complementary coverage of the lysosomal microenvironment in human neurons and mouse brains (Fig. 1D, E, F). Lysosomal membrane proteins such as vacuolar ATPase (v-ATPase) subunits [49], LAMP proteins, and Ragulator subunits [50] are identified and enriched by all three probes (Supplemental Figure S1). Lysosomal lumen proteins, especially hydrolases, are highly enriched in Lyso-IP proteomics, consistent with the degradative nature of the isolated organelles. Besides lysosome-resident proteins, both Lyso-APEX and Lyso-BAR proteomics captured dynamic lysosomal interaction partners related to organelle trafficking and axon transport (*e.g.*, Kinesins, MAPs) [44]. Lyso-APEX favored surface-bound and surface-interacting proteins over luminal proteins due to the limited membrane permeability of reactive phenol-biotin generated on the cytosolic face of lysosomes during APEX-mediated labeling (Fig. 1C, D). By contrast, Lyso-BAR revealed more intraluminal lysosomal proteins since BAR activation in fixed brain tissues requires membrane permeabilization. Lyso-BAR proteomics in mouse brain also captured numerous synaptic proteins, likely due to enhanced synaptic maturation in vivo compared to cultured iPSC-derived i^3^Neurons (Supplemental Figure S1). Collectively, combining Lyso-APEX, Lyso-IP, and Lyso-BAR proteomic strategies allows us to obtain comprehensive lysosomal lumen and membrane proteomes as well as lysosomal interactions in both cultured human i^3^Neurons and fixed mouse brains.

### Loss of progranulin results in upregulated vacuolar ATPases and elevated lysosomal pH in human neurons

**Fig. 2:**
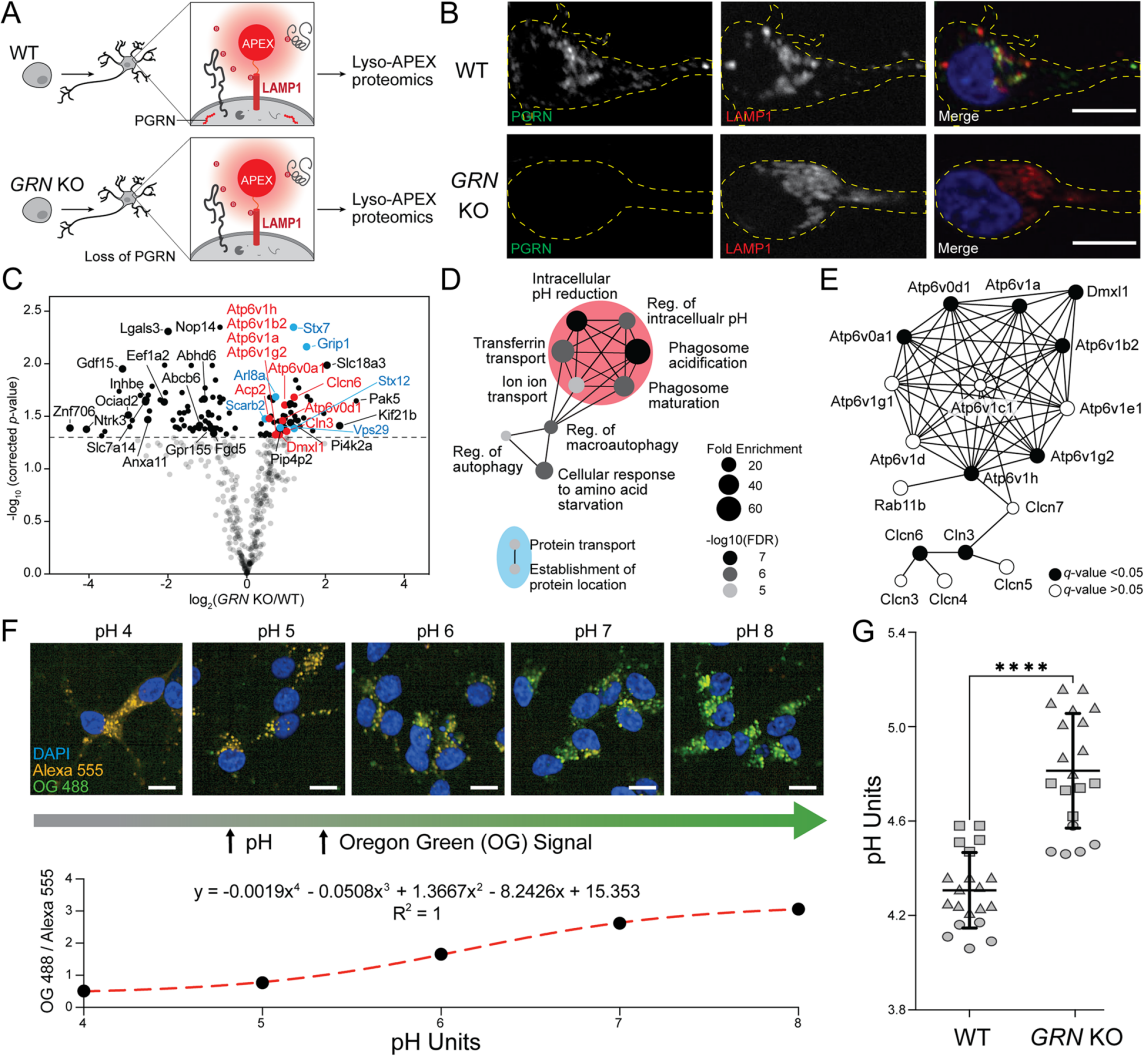
Progranulin-null human neurons have altered lysosomal membrane and interacting proteins and elevated lysosome pH. **A** Schematic of Lyso-APEX in WT and isogenic *GRN* KO i^3^Neurons. **B** Fluorescence imaging showing the colocalization of PGRN with lysosomes in WT i^3^Neurons and loss of PGRN signal in *GRN* KO i^3^Neurons. Scale bar is 10 μm. **C** Volcano plot of Lyso-APEX proteomics in *GRN* KO vs. WT i^3^Neurons (*N* = 4 for each group). Cytosolic enriched proteins and nonspecific labeling were removed from the volcano plot based on WT Lyso-APEX vs. Cytosolic-APEX comparison. Red and blue colored proteins belong to lysosomal pH and protein transport GO-terms, respectively. **D** GO enrichment analysis of significantly upregulated biological processes in *GRN* KO vs. WT Lyso-APEX proteomics. **E** Protein network analysis of increased vacuolar-ATPase subunits and their interactors in PGRN-null neurons. **F** Live cell ratiometric lysosome pH assay. pH calibration curve is generated based on the ratio of pH-sensitive Oregon Green-488 dextran signal and pH-insensitive/loading control Alexa Fluor-555 red dextran in WT i^3^Neurons. Scale bar is 10 μm. Other linear and nonlinear curve fitting models are provided in Supplementary Figure S2E. **G** Lysosome pH measurements in WT vs. *GRN* KO i.^3^Neurons; three independent experiments are represented with different shapes, each with 5–10 wells of neuron culture replicates (**** denotes *p*-value < 0.0001)

Equipped with these new tools, we characterized how progranulin loss altered lysosomal biology. Using CRISPR-Cas9, we knocked out the *GRN* gene in wild-type (WT) iPSCs harboring the Lyso-APEX probe and differentiated them into cortical neurons (Fig. 2A). Immunofluorescence microscopy showed that the PGRN protein colocalizes with lysosomes in WT i^3^Neurons, and that no PGRN signal was observed in *GRN* KO i^3^Neurons (Fig. 2B). Using Lyso-APEX proteomics, we found that PGRN depletion altered the abundance of many lysosome membrane proteins and lysosome interaction partners in human neurons (Fig. 2C). Gene Ontology (GO) enrichment analysis revealed upregulation of proteins related to lysosomal acidification and autophagy, including numerous v-ATPases (ATP6Vs) and chloride channel proteins (CLCNs) (Fig. 2D, E, Supplementary Figure S2A) [51]. GO enrichment analysis of significantly downregulated proteins indicated impaired lysosomal transport and RNA processing (Supplementary Figure S2B). Several lysosome proteins (Atp6v1g2, Clcn6, Tecpr1, Pld3) were increased in Lyso-APEX but decreased in cytosolic-APEX, indicating their potential translocation from the cytosol to the lysosome in PGRN-deficient neurons (Supplementary Figure S2C, D). Given the centrality of v-ATPases in establishing the acidic lysosomal lumen pH and the strong upregulation of acidification-related proteins in PGRN deficiency, we hypothesized that lysosomal pH could be perturbed by the loss of PGRN inside the neuronal lysosome.

To measure neuronal lysosomal acidification, we used a ratiometric fluorescent dextran assay. We co-generated an *in-situ* calibration curve using buffers of known pH, allowing accurate calculations of absolute pH within the lysosome with both nonlinear and linear curve fitting models (Fig. 2F, Supplementary Figure S2E). Lysosomal pH is significantly increased in *GRN* KO i^3^Neurons (4.81 ± 0.24) compared to WT i^3^Neurons (4.31 ± 0.16). While this difference in pH may seem like a subtle change, it equates to a nearly three-fold decrease in the concentration of protons in the lysosomal compartment of *GRN* KO i^3^Neurons compared to WT counterparts due to the logarithmic nature of the pH scale ([H^+^] in WT ≈ 52 ± 19 μM, *GRN* KO ≈ 18 ± 9 μM). These observations show that *GRN* KO i^3^Neurons have alkalinized lysosomes, which could trigger the upregulation of acidification machinery to compensate for this effect.

### Progranulin-null lysosomes contain increased hydrolases levels but have decreased enzymatic activity

**Fig. 3:**
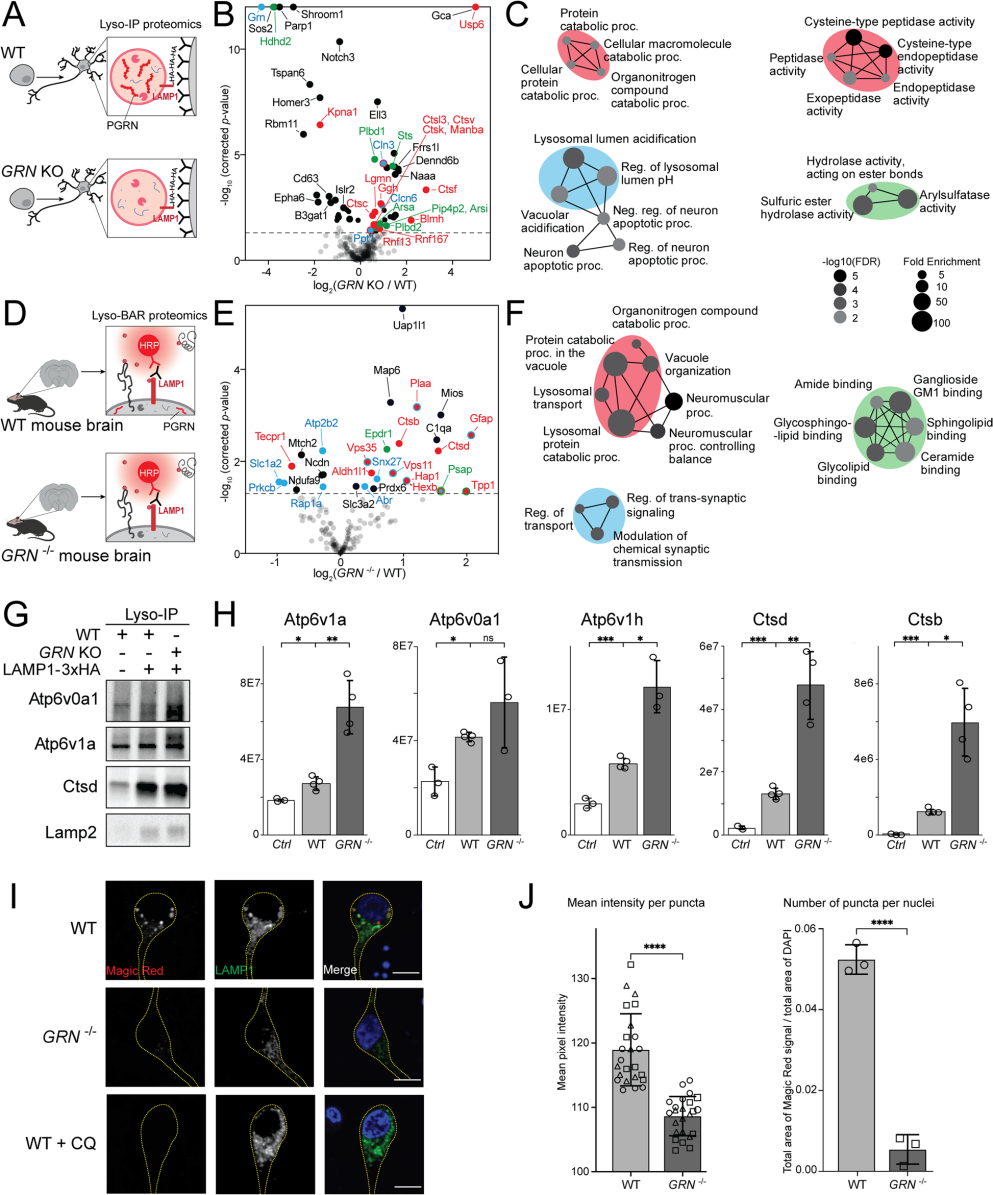
Progranulin-null lysosomes from human neurons and mouse brains contain increased hydrolases levels but have decreased enzymatic activity. **A** Schematic of intact lysosomal isolation (Lyso-IP) proteomics in *GRN* KO vs. WT i^3^Neurons. **B** Volcano plot of Lyso-IP proteomics showing protein changes related to protein catabolic processes (red), lysosomal pH (blue), and hydrolase activities (green). Nonspecific labeling proteins were removed from the plot based on WT Lyso-IP vs. control i^3^Neurons without HA expression. **C** GO enrichment analysis of significantly changed proteins in *GRN* KO vs. WT Lyso-IP proteomics (left: biological processes; right: molecular functions). Color code corresponds to the volcano plot. **D** Schematic of mouse brain Lyso-BAR labeling in GRN^−/−^ vs. WT mice. **E** Volcano plot showing Lyso-BAR protein changes in *GRN*^*−/−*^ vs. WT mouse brains after removing nonspecific labeling proteins. **F** GO enrichment analysis of significantly changed proteins in GRN ^−/−^ vs. WT Lyso-BAR proteomics. (**G**) Western blot analysis showing elevated V-ATPases and cathepsin D levels from isolated GRN KO vs. WT lysosomes in i^3^Neurons. **H** Targeted PRM protein quantification of v-ATPases and cathepsins from Lyso-BAR mouse brains. **I** Fluorescence imaging of Magic Red assay to measure cathepsin B enzymatic activity in i^3^Neurons. CQ stands for chloroquine treatment (50 μM for 24 h). Scale bar is 10 μm. **J** Quantification of absolute and relative fluorescence intensities indicate decreased cathepsin B activity in *GRN* KO vs. WT i^3^Neurons

Lysosomes require acidic luminal pH to degrade proteins using hydrolases [1]. Since lysosomes from progranulin-null neurons are less acidic, we hypothesized that these lysosomes may have altered abundances or activity of pH-dependent hydrolases. Using Lyso-IP proteomics, we characterized lysosome composition in *GRN* KO vs. WT i^3^Neurons (Fig. 3A). PGRN protein was confirmed to be enriched in isolated lysosomes and absent in *GRN* KO i^3^Neurons (Supplementary Figure S3A). Proteins involved in catabolism and lysosomal acidification were significantly increased in PGRN-deficient lysosomes in human neurons (Fig. 3B, C, Supplementary Figure S3C). To investigate the impact of progranulin deficiency on lysosomes in mouse brain, we conducted Lyso-BAR proteomics in *GRN*^−/−^ vs. WT fixed mouse brains (Fig. 3D). Similar protein catabolic processes were upregulated in *GRN*^−/−^ mice (Fig. 3E, F, Supplementary Figure S3E). Because Lyso-IP and Lyso-BAR probes have higher variations compared to Lyso-APEX probe (Fig. 1F), we conducted western blotting and targeted parallel reaction monitoring (PRM) assay to specifically quantify v-ATPases and cathepsin proteases in Lyso-IP and Lyso-BAR samples (Fig. 3G, H). V-ATPases and cathepsins showed consistent upregulation in lysosomes from PGRN-deficient i^3^Neurons and mouse brains.

Prior studies of *GRN*^−/−^ mouse models have suggested that cathepsins may be less active in progranulin-null cells despite increased abundance [30, 52, 53]. To directly evaluate the impact of progranulin depletion on lysosomal activity in human neurons, we quantified cathepsin B activity using a Magic Red assay in living WT and *GRN* KO i^3^Neurons. We observed a significant decrease in cathepsin B activity in PGRN-null i^3^Neurons compared to WT, indicating impaired proteolytic function (Fig. 3I, J, Supplementary Figure S3B). To mimic alkalinization-related phenotypes observed in *GRN* KO i^3^Neurons, we treated neurons with chloroquine, an agent that neutralizes lysosomal pH. As predicted, direct alkalinization of lysosomes with chloroquine treatment reduced Magic Red fluorescence (Fig. 3I). These findings confirm that although lysosomal hydrolases were upregulated in the absence of progranulin, their activity was decreased, likely due to alkalinized lysosomal lumens.

### Characterizing global protein turnover in human iPSC-derived neurons

**Fig. 4:**
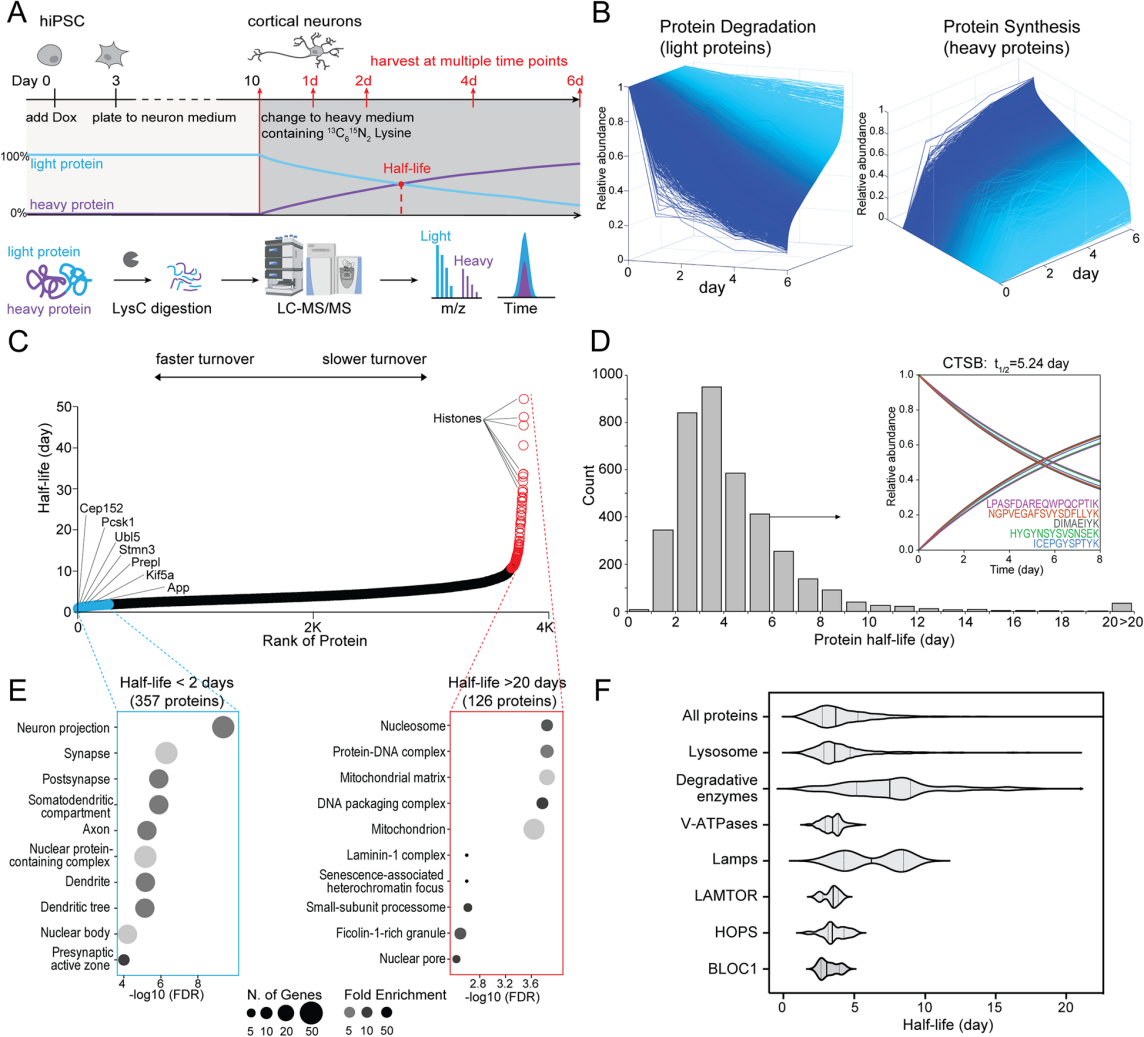
Measuring global protein half-lives in cultured human i^3^Neurons. **A** Schematic of dSILAC proteomics to measure global protein half-lives in cultured human i^3^Neurons. Cortical neurons were grown in normal medium until day 10 and then switched to heavy lysine-containing medium. Neurons are harvested at 1, 2, 4, and 6 days after medium switch followed by bottom-up proteomics. **B** Time-dependent changes of relative light and heavy protein abundances indicating protein degradation and synthesis processes, respectively, in WT i^3^Neurons. **C** Scatter plot of ranked protein half-lives measured in WT i^3^Neurons. **D** Histogram distribution of protein half-lives in WT i^3^Neurons. An example cathepsin B (CTSB) protein with five unique peptide sequences is illustrated in the inset. **E** GO enrichment analysis of the fast (left) and slow (right) turnover proteins in WT i^3^Neurons. **F** Violin plots of half-life distributions from lysosomal proteins in WT i^3^Neurons

Since lysosomes are major proteostatic organelles and their degradative function is impaired in progranulin-depleted neurons, we hypothesized that progranulin deficiency could influence global proteostasis. To measure the global protein turnover in neurons, we designed a dynamic SILAC proteomic method in cultured i^3^Neurons to calculate neuronal protein half-lives (Fig. 4A). By modeling the peptide degradation curves in WT i^3^Neurons, we found that most peptide degradations follow first-order exponential decay, consistent with other cell types in prior studies (Fig. 4B, Supplementary Figure S4A) [54]. Peptide level and protein level half-lives correlate well with each other, with a median half-life of around 4 days (Fig. 4C, D and Supplementary Figure S4B, C). Therefore, peptide and protein half-lives can be calculated using a single time point at 4 days (96 h) after heavy medium switch (Supplementary Figure S4D). As we examined the distribution of protein half-lives, we found that numerous histones, nucleoporin proteins (Nups), and inner mitochondrial membrane proteins have extremely long half-lives (> 20 days) in i^3^Neurons, in agreement with recent studies in primary rodent neurons and brain tissues [55–57]. In contrast, proteins related to neurosecretion (GPM6B, VGF), axonal transport (kinesins), and ubiquitination (UBL4, USP11) have very short half-lives (0.3–2 days) (Fig. 4E, Supplemental Figure S4B). Notably, one of the shortest half-life proteins in the entire neuronal proteome was STMN2, a microtubule-binding, Golgi-localized protein implicated in ALS pathogenesis [58, 59]. Lysosomal proteins have a median half-life of 3.6 days, slightly shorter than the median half-life of global neuronal proteins. Further investigation into the lysosomal compartment revealed a median half-life of 7.5 days for degradative enzymes, 3.5 days for v-ATPases, 6.2 days for lysosome-associated membrane glycoproteins (Lamps), 3.5 days for LAMTOR and HOPS complex subunits, and 3.1 days for BLOC1 complex subunits (Fig. 4F). Together, this method enabled us to calculate global protein half-lives in living human i^3^Neurons for the first time.

### Loss of progranulin alters neuronal protein turnover and decreases lysosomal degradative function

**Fig. 5:**
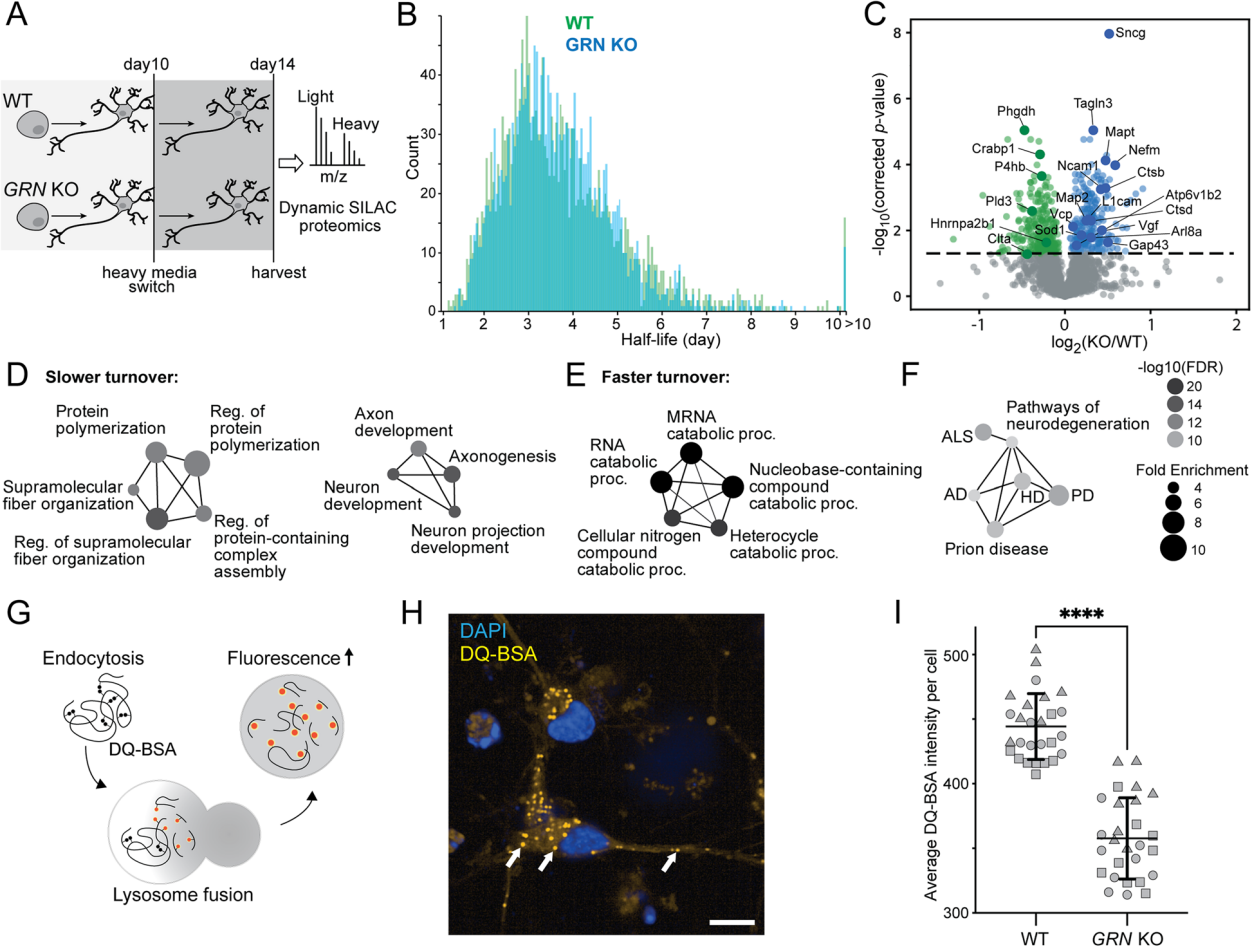
Global protein turnover and lysosomal degradative function are impaired in progranulin-null human neurons. **A** Schematic of protein half-life measurements in *GRN* KO vs. WT i^3^Neurons using dynamic SILAC proteomics. **B** Histogram distribution of global protein half-lives in *GRN* KO (blue) vs. WT (green) i^3^Neurons. **C** Volcano plot of protein half-life changes in *GRN* KO vs. WT i^3^Neurons. **D** GO enrichment analysis of biological processes from proteins with significantly slower turnover. **E** GO enrichment analysis of biological processes from proteins with significantly faster turnover. **F** KEGG pathways enriched from significantly altered protein half-lives in *GRN* KO vs. WT i^3^Neurons. **G** Schematic of the DQ-BSA Red assay to measure lysosomal degradative function. Extracellular DQ-BSA with self-quenched dye is endocytosed into i^3^Neurons and trafficked to the lysosome, where it is degraded into smaller protein fragments with isolated fluorophores with fluorescence signals. **H** Representative fluorescence imaging of DQ-BSA Red assay showing DQ-positive lysosomes in i^3^Neurons. Scale bar is 10 μm. **I** Quantification of the fluorescence intensities of the DQ-BSA Red assay in WT vs. *GRN* KO i^3^Neurons, normalized to the total number of puncta in two groups

Using our dynamic SILAC proteomics approach, we evaluated protein turnover in WT vs. *GRN* KO i^3^Neurons (Fig. 5A). The median of global protein half-lives remained unchanged, but a remarkable 25% of all measured proteins presented significantly altered half-lives in *GRN* KO vs. WT i^3^Neurons (corrected *p*-value < 0.05) (Fig. 5B and C). Because protein half-life measurements have much smaller fold changes compared to protein abundance levels, we did not use any fold change ratio cutoff. Proteins related to polymerization and fiber organization showed significantly slower turnover, which may indicate a propensity for protein misfolding and aggregation in *GRN* KO neurons related to FTD pathogenesis (Fig. 5D) [60]. Despite the significantly slower turnover of both cathepsin B and cathepsin D, proteins related to RNA catabolic processes showed faster turnover, which further implicates the disturbance of molecular degradation pathways (Fig. 5E). Go enrichment analysis from altered proteins showed enrichment in ALS/FTD and other neurodegenerative diseases, suggesting potential converging pathways among different neurodegenerative diseases and dysfunction of key regulators of proteostasis (Fig. 5F).

Given our observations that lysosomes within *GRN* KO i^3^Neurons are alkalinized, have reduced cathepsin activity, and exhibit substantial changes in global protein homeostasis, we predicted that *GRN* KO lysosomes would exhibit impaired lysosome-mediated protein degradation. We directly assayed lysosomal degradative capacity using a fluorescent DQ-BSA Red assay (Fig. 5G, H) [61, 62]. The DQ-BSA substrate is initially self-quenching due to the close spatial proximity of the fluorophores. Once cleaved in acidic lysosomes, the DQ-BSA substrate exhibits bright fluorescence signals. The mean DQ-BSA intensity in *GRN* KO i^3^Neurons was significantly decreased compared to WT neurons (Fig. 5I), similar to pharmacological inhibition of lysosomal degradation using chloroquine (Supplemental Figure S5A). The change in active proteolysis was independent of lysosomal biogenesis, as there was no change in the number of puncta per cell in *GRN* KO vs. WT (Supplemental Figure S5B). Taken together, these results show that *GRN* KO lysosomes have significantly hindered proteolytic capacity, consistent with our observations of pathological impairment in lysosomal acidification and impaired lysosomal hydrolase activity.

### An isogenic series of FTD patient-derived i3Neurons with deficient PGRN exhibit altered protein homeostasis

**Fig. 6:**
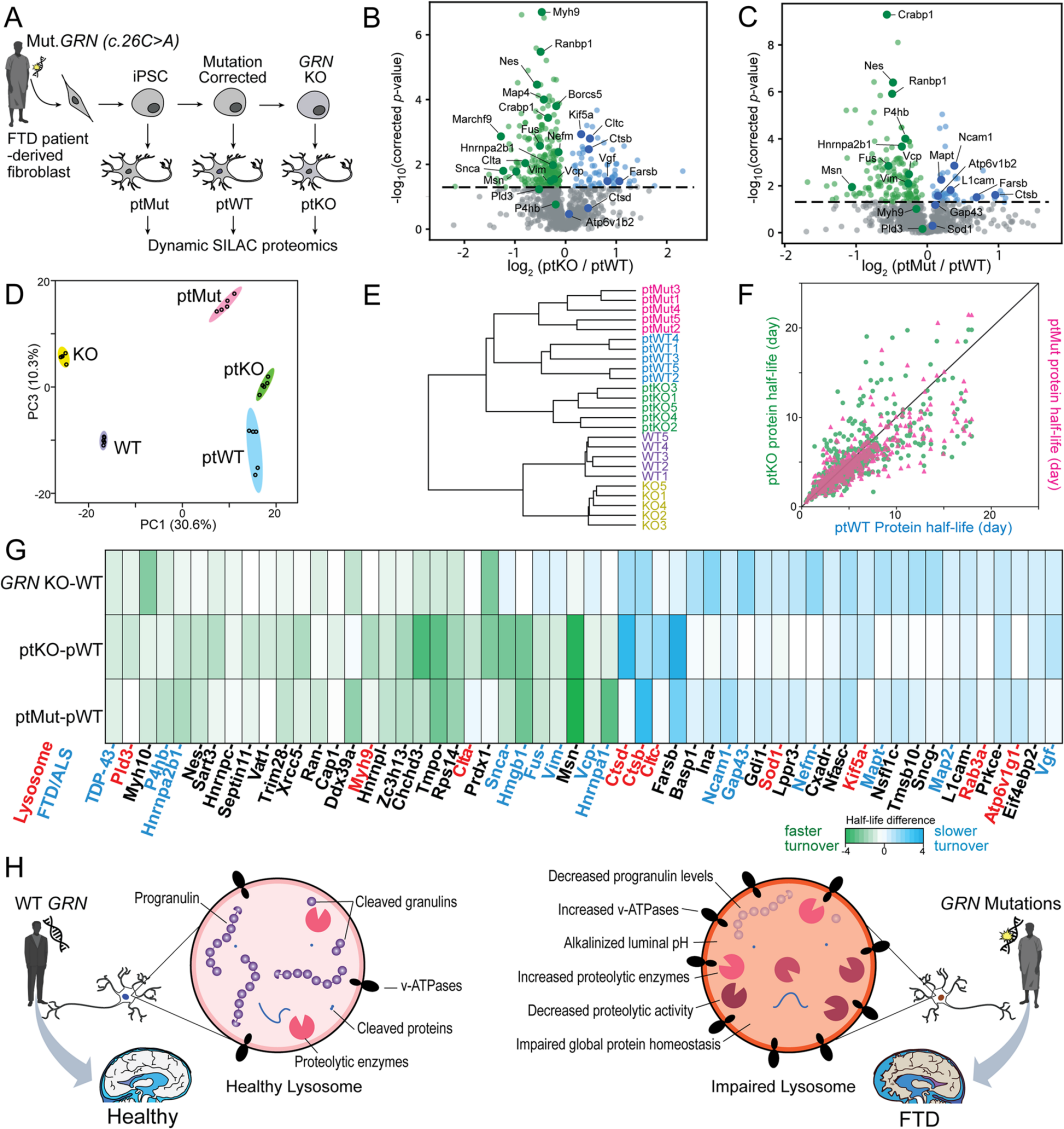
FTD patient-derived i^3^Neurons with mutant *GRN* reveal altered protein turnover of lysosomal enzymes and FTD-associated proteins. **A** Generation of a set of FTD patient fibroblast-derived i^3^Neurons. First, CRISPR-Cas9 was used to insert an inducible *NGN2* cassette into the *AAVS1* locus of a patient fibroblast-derived iPSC line (ptMut). Next, CRISPR-Cas9 was used to correct the *GRN* mutation in ptMut to create an isogenic control iPSC line (ptWT) and then to knockout *GRN* in pWT to create the ptKO iPSC line. These iPSC lines were then differentiated into i^3^Neurons and dSILAC proteomics was performed. **B** Volcano plot of protein half-life changes in ptKO vs. ptWT i^3^Neuron. **C** Volcano plot of protein half-life changes in ptMut vs. ptWT i^3^Neuron. **D** Principal component analysis using protein half-lives in GRN-KO, WT, ptKO, ptMut, and ptWT i^3^Neurons groups. **E** Hierarchical clustering of five i^3^Neurons groups with five biological replicates in each group. **F** Scatter plot of protein half-life changes in ptKO vs. ptWT and ptMut vs. ptWT comparisons showing the consistency and potential gene dosage effect of ptKO and ptMut i^3^Neurons. **G** Heatmap showing key overlapping protein turnover changes in *GRN* KO vs. WT, ptKO vs. ptWT, and ptMut vs. ptWT i^3^Neurons. Heatmap colors represent the absolute half-life differences in days between comparison groups. Key proteins from lysosomes and relevant to FTD/ALS are highlighted in red and blue, respectively. **H** Schematic of proposed lysosomal impairment in progranulin-deficient neurons caused by GRN mutations in FTD patients

To further explore the relationship between *GRN* insufficiency and protein homeostasis abnormalities, we created i^3^Neurons from an FTD patient-derived iPSC line with a heterozygous *GRN* mutation [35] (c.26 C > A, p.A9D; referred subsequently as ptMut), as well as the isogenic iPSC control line with corrected *GRN* mutation (ptWT). We further knocked out *GRN* in this control line to create an additional isogenic *GRN* KO iPSC line (ptKO) (Fig. 6A, Table 1). After differentiating each iPSC line to i^3^Neurons, performing dSILAC, and measuring protein half-lives, we found that over 25% of proteins showed significantly altered half-lives (corrected *p*-value < 0.05) in ptKO compared to ptWT i^3^Neurons (Fig. 6B), consistent with *GRN*-KO vs. WT comparison in Fig. 5C. About 15% of protein half-lives were significantly altered in ptMut compared to ptWT group (Fig. 6B and C). Principal component analysis and hierarchical clustering showed complete separations of both genetic background and *GRN* genotypes from five i^3^Neurons lines (*GRN*-KO, WT, ptKO, ptMut, ptWT) based on protein half-lives (Fig. 6D, E). The overall protein half-life changes also suggested a potential gene dosage effect, in which many proteins have greater fold changes in *GRN*-KO neurons compared to *GRN*-mutant neurons (Fig. 6F, Supplementary Figure S6A). Half-life changes of key overlapping proteins in the three comparisons (*GRN*-KO vs. WT, ptKO vs. ptWT, ptMut vs. ptWT) are highlighted in Fig. 6G and Supplementary Figure S6B.

The findings in patient-derived *GRN* mutant and KO neurons validate our observations of dysregulated protein homeostasis in settings of *GRN* depletion and insufficiency, including alterations in the half-lives of numerous neurodegeneration-associated proteins. Many lysosomal enzymes showed prolonged protein half-lives, such as cathepsins (CTSD, CTSB), which was especially notable given our direct measurements of increased cathepsin levels and reduced CTSB activity in *GRN* KO neurons. Our findings additionally show that substantial upregulation of numerous lysosomal-associated proteins and enzymes occurs in *GRN*-deficient neurons – many via prolongation in protein half-lives – but that these homeostatic changes are insufficient to normalize lysosomal degradative capacity. A most recent study also demonstrated the elevated lysosome pH in FTD patient-derived neurons which agrees well with our findings [63]. As summarized in Fig. 6H, we propose that GRN mutations that cause PGRN deficiency inside neuronal lysosomes result in alkalinized lysosomal pH, decreased proteolytic activities, and impaired global protein homeostasis that eventually lead to frontotemporal dementia.

## Discussions

Lysosomal dysfunction is a convergent pathological mechanism across multiple neurodegenerative diseases [5, 6]. Progranulin, a glycoprotein linked to FTD, ALS, PD, and AD, is trafficked to, processed by, and resides within the lysosome [16]. Despite this knowledge, the primary molecular functions of progranulin and the impact of progranulin deficiency on lysosomal biology and protein homeostasis remain unclear. This is in part due to limited tools available for understanding the role of progranulin in the highly dynamic lysosomes in the brain. Here, we designed a combination of in vitro and in situ proximity labeling, lysosome immunopurification, and dynamic SILAC proteomic approaches to map the organellar and cellular architectures of neuronal progranulin deficiency.

For the first time, we implemented the antibody-guided biotinylation strategy to measure lysosomal composition in the brain and the lysosomal immunopurification method to characterize neuronal lysosomes. We additionally developed a neuron dynamic SILAC proteomic method to calculate protein half-lives in i^3^Neurons for the first time. Despite the application of dynamic SILAC in various cell culture and mouse models, it remains challenging to measure protein turnover rates in non-dividing cells, particularly in human neurons [55, 56, 64]. Many neuronal proteins exhibit extremely long half-lives, particularly nuclear proteins, due to a lack of cell division. We measured the global protein turnover in i^3^Neurons with a medium half-life of ~ 4 days and found that the dynamics of most proteins can be modeled using first-order exponential decay. This enabled the measurement of global neuron protein half-lives using a 4-day single time point method, significantly reducing the starting materials and reagents compared to the multiple-time-point method and allowing the streamlined comparison of multiple i^3^Neuron lines with different genome backgrounds and *GRN* genotypes.

Using these new multi-modal proteomic strategies, we discovered that progranulin deficiency leads to increased expression of v-ATPases on the lysosomal membrane in i^3^Neurons. V-ATPases consist of two domains: the stationary membrane domain V0 and the cytosolic-facing rotatory domain V1 [65]. Loss of PGRN may recruit more cytosolic facing rotatory domain V1 to the lysosome but impede the attachment of the V1 domain to the stationary membrane V0 domain to allow for the proper proton transport across the lysosome membrane [41]. Upon further investigation, we discovered that progranulin deficiency had a severe impact on the lysosomes’ ability to properly acidify, which results in impaired hydrolytic activity despite the upregulation of acidification machinery. These results suggest that progranulin plays an important role in maintaining lysosomal pH, with v-ATPases either contributing to that effect or providing a compensatory response for that effect. Since alkalinized lysosomes cannot properly hydrolyze substrates, we next looked at how the contents of progranulin-null lysosomes were affected. We found that several lysosomal enzymes were upregulated both in the mouse and human datasets, notably cathepsins. We showed decreased cathepsin B activity in live neurons, a phenomenon only shown in in vitro assays before [35, 66–68]. Similar perturbations of lysosomal acidification have been reported in non-neuron cells and other neurodegenerative diseases [69, 70].

Mutations in the *GRN* gene cause progranulin deficiency inside the lysosome and have been shown to impair lysosomal function and the autophagy pathway [13, 18]. However, whether progranulin deficiency alters protein turnover in human neurons has not been systematically investigated previously. We found that progranulin deficiency broadly influenced proteostasis, altering the half-lives of over 15% and 25% neuron proteins in *GRN* mutant and KO i^3^Neurons, respectively. Lysosome degradative capacity was compromised by PGRN deficiency, as evidenced in our DQ-BSA assay. Critically, the recapitulation of global proteostasis defects in FTD-patient-derived neurons suggests that altered protein turnover rates are relevant to disease pathophysiology.

Although we have established exciting new tools and characterized the neuronal lysosome quite extensively, there are several limitations in this study. LAMP1 is a classic lysosome marker, but it is also expressed on late endosomes and other endocytic species [71]. Despite this limitation, our data is consistent with degradative lysosome proteomics, and we obtained new insights into neuronal lysosomes specifically. We also recognize that human iPSC-derived neurons are not fully mature and representative of late-stage disease, and therefore have supplemented i^3^Neuron data with lysosomal proteomics in aged mice. As neurons are the major cell type of the brain, Lyso-BAR proteomics provides consistent and complementary lysosomal changes compared to cultured i^3^Neuron. However, Lyso-BAR method is not cell-type specific and will also include lysosome profiles from other cell types, such as microglia, the primary immune cells in the brain, which has higher expression level of progranulin compared to neurons [9, 15]. Indeed, several proteins related to innate immunity were significantly increased in PGRN-deficient mouse brain (C1QA, C1QB, GFAP), implicating progranulin’s role in regulating neuroinflammation [52, 72]. But further investigations are needed to study human microglial lysosomes and compare them to neuronal lysosomes in the context of PGRN deficiency. How PGRN loss specifically influences v-ATPases, through complex assembly, structure, membrane topology, or protein bindings, also needs to be further investigated in future studies. Our ongoing efforts also aim to increase the proteome coverage and quantification accuracy for protein half-life measurements in i^3^Neurons. Furthermore, future research can focus on individual proteins with altered lysosomal enrichment and half-lives as novel handles for elucidating disease mechanisms and discovering disease biomarkers. Future studies can assess whether these neuronal proteostatic changes manifest in established mechanisms of neurodegenerative pathology, such as stress granule persistence, impaired macroautophagy, and failed fusion of lysosomes to autophagosomes.

## Conclusions

Overall, this study developed and implemented a set of novel proteomic techniques to decipher neuronal lysosomal biology and proteostasis in the context of PGRN deficiency that causes frontotemporal dementia. We provided new insights into progranulin function in regulating lysosomal pH, lysosomal catabolic activity, and global proteostasis in neurons, opening numerous avenues for future follow-up studies to determine specific molecular mechanisms underpinning the protein changes discovered here. This work also illustrated a roadmap for how multi-modal proteomics can be used to illuminate lysosomal biology, providing useful tools and data resources that can be applied to characterize other organelle dynamics in neurons.

### Supplementary Information

**Additional file 1: Supplementary Figure S1.** Confirmation of the Lyso-IP, Lyso-BAR, and Lyso-APEX probe locations at the lysosome, related to Figure 1. (A) Fluorescence imaging of Lyso-APEX i^3^Neurons and negative control neurons without H_2_O_2_ treatment. Biotinylation cloud stained with streptavidin (SA-680), colocalizes with the bait protein LAMP1. Negative control group does not have SA signal. Nuclei were stained by Hoechst. Scale bar is 10 μm. (B) Western blot analysis of isolated lysosomes from Lyso-IP i^3^Neurons compared to whole cell lysate and negative control without HA expression. (C) Protein network analysis of Lyso-IP enriched known lysosome proteins from i^3^Neurons. (D) Protein network analysis showing enriched lysosome, vesicle-mediated transport, and synapse processes from Lyso-Bar enriched proteins in mouse brains compared to Lyso-APEX enriched proteins in i^3^Neurons. (E) Venn diagram comparison of significantly enriched proteins in Lyso-APEX, Lyso-IP, and Lyso-BAR proteomics. **Supplementary Figure S2.** Proximity labeling proteomics and lysosome pH measurement in progranulin-null i^3^Neurons, related to Figure 2. (A) GO enrichment analysis of significantly upregulated molecular functions in GRN KO vs. WT Lyso-APEX proteomics. (B) GO enrichment analysis of significantly downregulated molecular functions in GRN KO vs. WT Lyso-APEX proteomics. (C) Volcano plot of cytosolic-APEX proteomics in GRN KO vs. WT i^3^Neurons (*N*=4 for each group). (D) Scatter plot of significantly changed proteins (corrected *p*-value <0.05) in both Lyso-APEX and cytosolic-APEX proteomics showing potential protein translocation in neurons. (E) Lysosomal pH measurements in WT vs. GRN KO i^3^Neurons with linear (left) and 3^rd^ order (right) calibration curve fitting. **Supplementary Figure S3.** Loss of progranulin results in elevated levels of lysosomal catabolic enzymes and decreased cathepsin B activity in human i^3^Neurons and mouse brains, related to Figure 3. (A) PGRN is enrichment in isolated WT lysosomes and is absent in GRN KO lysosomes from Lyso-IP proteomics data. (B) Additional replicate of Magic Red assay showing reduced cathepsin B activity in GRN KO i^3^Neurons compared to WT. Scale bar is 10 μm. (C) Volcano plot of Lyso-IP proteomics in KO vs. WT i^3^Neurons without filtering, related to Figure 3B (*N*=4 for each group). (D) Protein network analysis of selected lysosome and synaptic proteins that are significantly changed in GRN KO vs. WT Lyso-IP proteomic. (E) Volcano plot of Lyso-BAR proteomics in GRN^-/-^ vs. WT mouse brains without filtering, related to Figure 3E (*N*=4 for each group). (**F**) Protein network analysis of selected lysosome and synaptic proteins that are significantly changed in GRN^-/-^ vs. WT Lyso-BAR proteomics. **Supplementary Figure S4.** Developing dynamic SILAC proteomics in i^3^Neurons to measure global neuron protein half-lives, related to Figure 4. (A) Histogram distribution of peptide level curve fitting R^2^ to first-order exponential decay. An example of Tau peptide degradation and synthesis curves is shown in the inset. (B) Scatter plot of ranked peptide level half-lives in WT i^3^Neurons. (C) Histogram distribution of peptide half-lives, consistent with protein level results in Figure 4D. (D) Scatter plot showing strong correlation of protein/peptide half-lives measured by multiple-time-point method (1, 2, 4, 6 days) and single-time-point method (4 day). **Supplementary Figure S5.** DQ-BSA Red Assay to measure lysosomal degradative function, related to Figure 5. (A) Chloroquine treatment (30 µM for 12 hours) impairs the lysosomal degradative function with significantly reduced DQ-BSA signals compared to untreated WT i^3^Neurons (*** denotes *p*-value < 0.001). (B) WT and GRN KO i^3^Neurons have similar total number of DQ-BSA fluorescent puncta, indicating similar overall levels of endocytosis and lysosomal biogenesis, related to Figure 5I. **Supplementary Figure S6.** Protein half-life changes caused by progranulin deficiency in GRN-KO, WT, ptKO, ptMut, and ptWT i^3^Neurons, related to Figure 6. (A) Scatter plot showing potential gene dosage effect of protein half-life changes in ptKO vs. ptWT and ptMutant vs. ptWT i^3^Neurons. (**B**) Heatmap showing overlapping protein half-lives in WT, GRN-KO, ptWT, ptKO, and ptMut i^3^Neurons. Heatmap colors represent the absolute half-life measurements in days.

**Additional file 2: Supplementary Table S1.** List of antibodies used in this study.

**Additional file 3: Supplementary Table S2.** Proteomics and statistical results for Lyso-APEX, Lyso-IP, and Lyso-BAR. Source data for Figs. 1, 2 and 3.

**Additional file 4: Supplementary Table S3.** Global protein half-lives and statistical results for WT, GRN-KO, ptKO, ptMut, and ptWT i^3^Neurons. Source data for Figs. 4, 5 and 6.

## Data Availability

All proteomics RAW files have been deposited in the PRIDE database (ProteomeXchange Consortium) with the data identifier PXD040251. All other supporting data are available within the article and the supplementary files.

## Abbreviations

PGRN: Progranulin
iPSC: Human induced pluripotent stem cell
dSILAC: Dynamic stable isotope labeling by amino acids in cell culture
FTD: Frontotemporal dementia
NCL: Neuronal ceroid lipofuscinosis
AD: Alzheimer’s disease
PD: Parkinson’s disease
Lyso-APEX: Lysosome engineered ascorbate peroxidase
Lyso-IP: Lysosome immunopurification
Lyso-BAR: Lysosome biotinylation by antibody recognition
GO: Gene ontology
WT: Wild-type
KO: Knock out
PFA: Paraformaldehyde
NGN2: Neurogenin2
SA: Streptavidin
FBS: Fetal bovine serum
