# Correction: Multi-modal proteomic characterization of lysosomal function and proteostasis in progranulin-deficient neurons

**DOI:** 10.1186/s13024-023-00696-3

**Published:** 2023-12-18

**Authors:** Saadia Hasan, Michael S. Fernandopulle, Stewart W. Humble, Ashley M. Frankenfield, Haorong Li, Ryan Prestil, Kory R. Johnson, Brent J. Ryan, Richard Wade-Martins, Michael E. Ward, Ling Hao

**Affiliations:** 1 National Institute of Neurological, Disorders and Stroke (NINDS), National Institutes of Health (NIH), Bethesda, MD USA; 2 Department of Neurodegenerative Disease, UK Dementia Research Institute, Institute of Neurology, University College London, London, UK; 3 Augusta University, University of Georgia Medical Partnership, Athens, GA USA; 4 Cambridge Institute for Medical Research, University of Cambridge, Cambridge, UK; 5 Medical Scientist Training Program, Feinberg School of Medicine, Northwestern University, Chicago, IL USA; 6 Department of Physiology, Anatomy and Genetics, Oxford Parkinson’s Disease Centre, Kavli Institute for Nanoscience Discovery, University of Oxford, Dorothy Crowfoot Hodgkin Building, South Parks Road, OX1 3QU Oxford, UK; 7 Department of Chemistry, George Washington University, Washington, DC USA

**Molecular Neurodegeneration (2023) 18:87**

10.1186/s13024-023-00673-w

The original article [1] contained an erroneous duplication of Fig. 1 over Fig. 3 during proofing. The corrected Fig. 3 has since been restored in the original article.
