# Supplementary material for: Multi-modal proteomic characterization of lysosomal function and proteostasis in progranulin-deficient neurons
Source: Mol Neurodegener. 2023 Nov 16;18:87. doi: 10.1186/s13024-023-00673-w (PMC10655356; doi:10.1186/s13024-023-00673-w)
Supplement: Supplementary file 1 — Additional file 1: Supplementary Figure S1. Confirmation of the Lyso-IP, Lyso-BAR, and Lyso-APEX probe locations at the lysosome, related to Figure 1. (A) Fluorescence imaging of Lyso-APEX i3Neurons and negative control neurons without H2O2 treatment. Biotinylation cloud stained with streptavidin (SA-680), colocalizes with the bait protein LAMP1. Negative control group does not have SA signal. Nuclei were stained by Hoechst. Scale bar is 10 μm. (B) Western blot analysis of isolated lysosomes from Lyso-IP i3Neurons compared to whole cell lysate and negative control without HA expression. (C) Protein network analysis of Lyso-IP enriched known lysosome proteins from i3Neurons. (D) Protein network analysis showing enriched lysosome, vesicle-mediated transport, and synapse processes from Lyso-Bar enriched proteins in mouse brains compared to Lyso-APEX enriched proteins in i3Neurons. (E) Venn diagram comparison of significantly enriched proteins in Lyso-APEX, Lyso-IP, and Lyso-BAR proteomics. Supplementary Figure S2. Proximity labeling proteomics and lysosome pH measurement in progranulin-null i3Neurons, related to Figure 2. (A) GO enrichment analysis of significantly upregulated molecular functions in GRN KO vs. WT Lyso-APEX proteomics. (B) GO enrichment analysis of significantly downregulated molecular functions in GRN KO vs. WT Lyso-APEX proteomics. (C) Volcano plot of cytosolic-APEX proteomics in GRN KO vs. WT i3Neurons (N=4 for each group). (D) Scatter plot of significantly changed proteins (corrected p-value <0.05) in both Lyso-APEX and cytosolic-APEX proteomics showing potential protein translocation in neurons. (E) Lysosomal pH measurements in WT vs. GRN KO i3Neurons with linear (left) and 3rd order (right) calibration curve fitting. Supplementary Figure S3. Loss of progranulin results in elevated levels of lysosomal catabolic enzymes and decreased cathepsin B activity in human i3Neurons and mouse brains, related to Figure 3. (A) PGRN is enrichment in isolated WT [file 13024_2023_673_MOESM1_ESM.pdf]

Multi-modal Proteomic Characterization of Lysosomal Function and Proteostasis in Progranulin-Deficient Neurons

Saadia Hasan[1,2,3#] (saadia.hasan@nih.gov), Michael S. Fernandopulle[1,4,5#] (michael.fernandopulle@nih.gov), Stewart W. Humble[1,6#] (stewart.humble@dpag.ox.ac.uk), Ashley M. Frankenfield[7] (afrankenfield@gwu.edu), Haorong Li[7] (haorong@gwu.edu), Ryan Prestil[4] (ryanprestil@gmail.com), Kory R. Johnson[1] (johnsonko@ninds.nih.gov), Brent J. Ryan[6] (brent.ryan@dpag.ox.ac.uk), Richard Wade-Martins[6] (richard.wade-martins@dpag.ox.ac.uk), Michael E. Ward[1,*] (wardme@nih.gov), Ling Hao[7,*] (linghao@gwu.edu)

[1]National Institute of Neurological Disorders and Stroke (NINDS), National Institutes of Health (NIH), Bethesda, MD, USA.

[2]UK Dementia Research Institute, Department of Neurodegenerative Disease, Institute of Neurology, University College London, London, UK.

[3]MD-PhD program, Augusta University/University of Georgia Medical Partnership, Athens, GA, USA.

[4]Cambridge Institute for Medical Research, University of Cambridge, Cambridge, UK.

[5]Medical Scientist Training Program, Feinberg School of Medicine, Northwestern University, Chicago, IL, USA.

[6]Oxford Parkinson's Disease Centre, Kavli Institute for Nanoscience Discovery, Department of Physiology, Anatomy and Genetics, Dorothy Crowfoot Hodgkin Building, University of Oxford, South Parks Road, Oxford, OX1 3QU UK.

[7]Department of Chemistry, George Washington University, Washington, DC, USA.

[#]Contributed equally to this paper

*Co-corresponding authors:

Michael E. Ward

Investigator, NIH/NINDS

wardme@nih.gov

Ling Hao

Assistant Professor, George Washington University

linghao@gwu.edu

Supplementary Figures

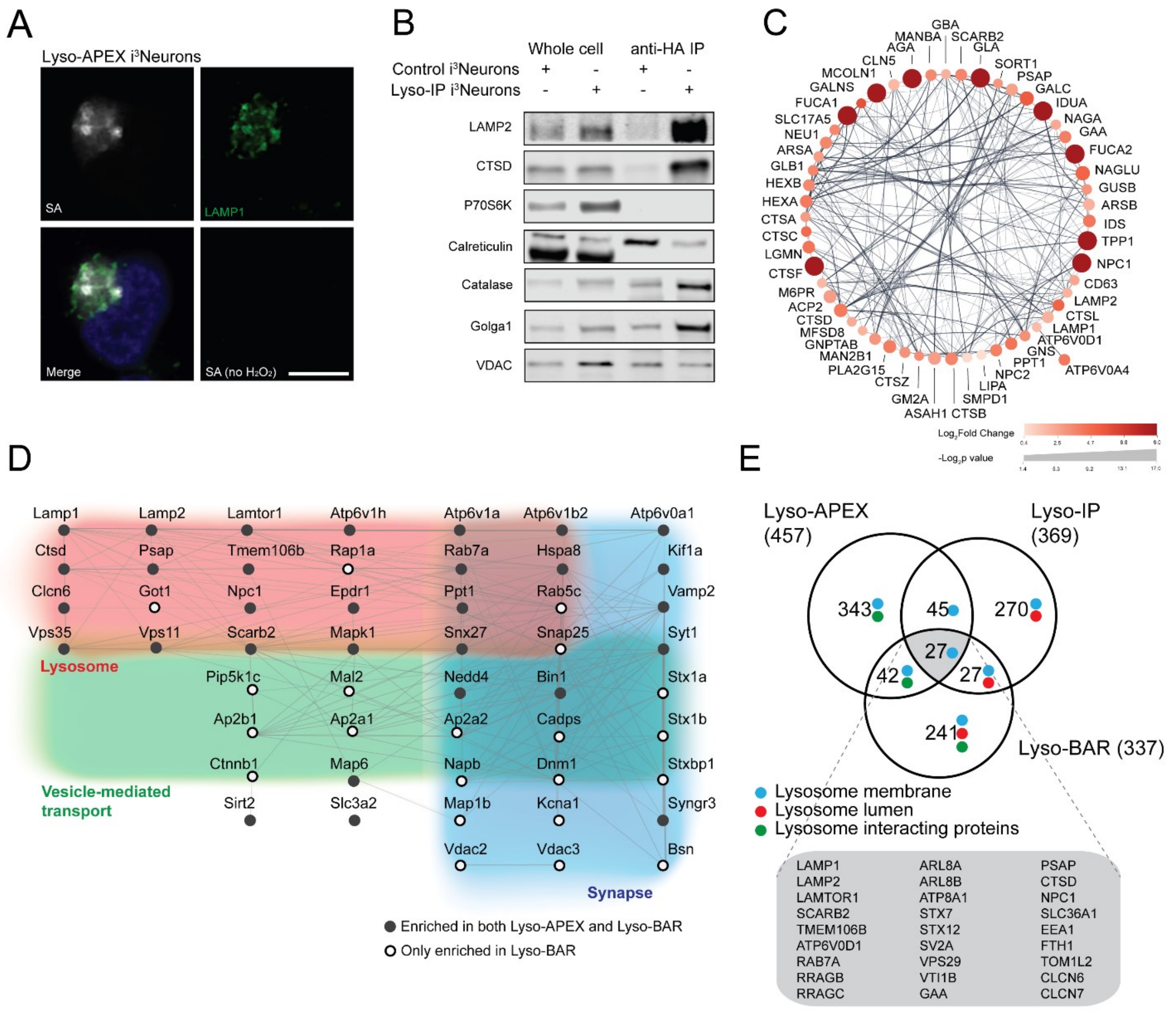

Supplementary Figure S1. **Confirmation of the Lyso-IP, Lyso-BAR, and Lyso-APEX probe locations at the lysosomes, related to Figure 1.** (**A**) Fluorescence imaging of Lyso-APEX i[3]Neurons and negative control neurons without H_2O_2 treatment. Biotinylation cloud stained with streptavidin (SA-680), colocalizes with the bait protein LAMP1. Negative control group does not have SA signal. Nuclei were stained with Hoechst. Scale bar is 10 μm. (B) Western blot analysis of isolated lysosomes from Lyso-IP i[3]Neurons compared to whole cell lysate and negative control without HA expression. (**C**) Protein network analysis of Lyso-IP enriched known lysosome proteins from i[3]Neurons. (**D**) Protein network analysis showing enriched lysosome, vesicle-mediated transport, and synapse processes from Lyso-Bar enriched proteins in mouse brains compared to Lyso-APEX enriched proteins in i[3]Neurons. (**E**) Venn diagram comparison of significantly enriched proteins in Lyso-APEX, Lyso-IP, and Lyso-BAR proteomics.

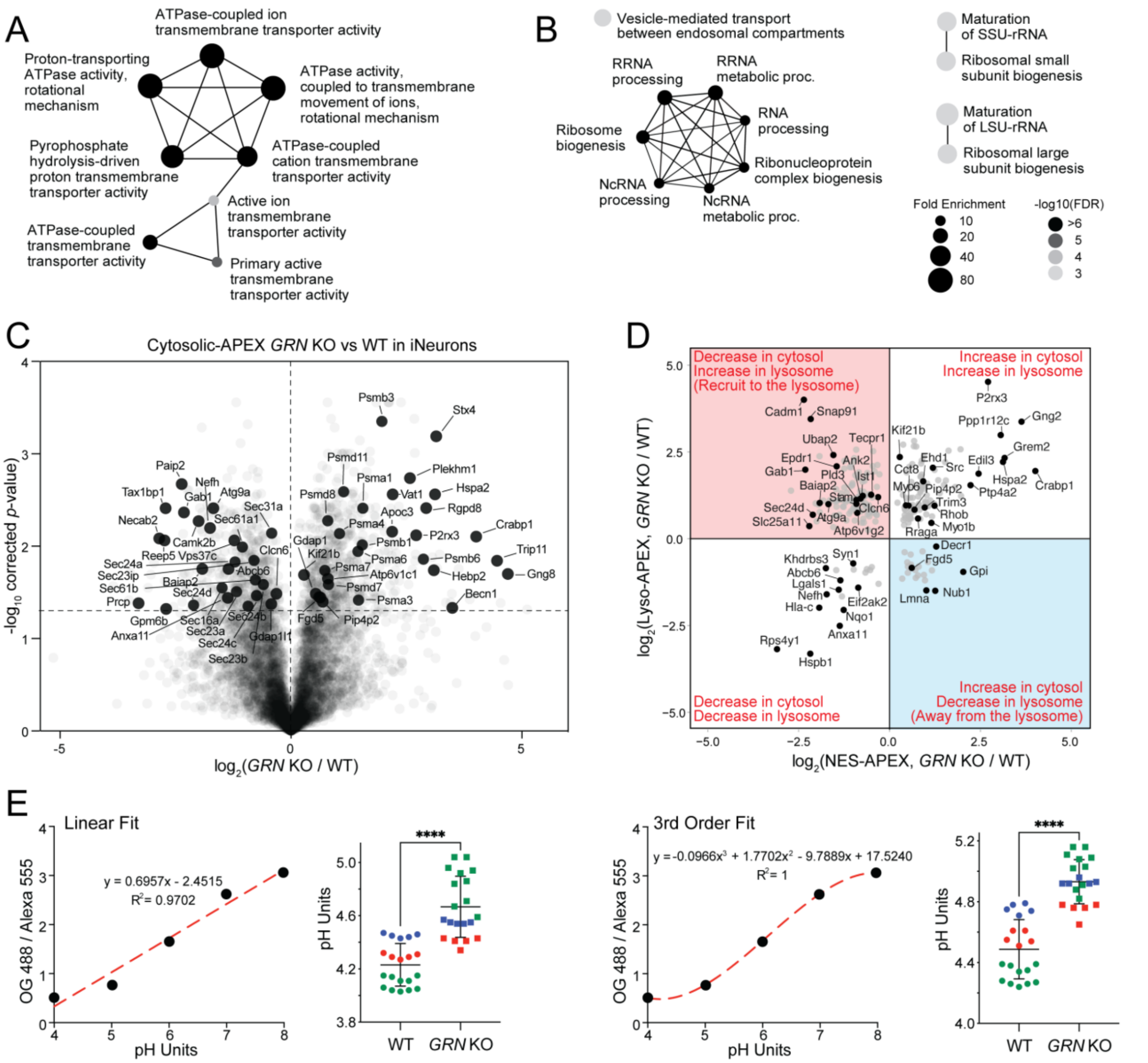

Supplementary Figure S2. Proximity labeling proteomics and lysosome pH measurement in progranulin-null i³Neurons, related to Figure 2. (A) GO enrichment analysis of significantly upregulated molecular functions in *GRN* KO vs. WT Lyso-APEX proteomics. **(B)** GO enrichment analysis of significantly downregulated molecular functions in *GRN* KO vs. WT Lyso-APEX proteomics. **(C)** Volcano plot of cytosolic-APEX proteomics in *GRN* KO vs. WT i³Neurons. **(D)** Scatter plot of significantly changed proteins (corrected *p*-value <0.05) in both Lyso-APEX and cytosolic-APEX proteomics showing potential protein translocation in neurons. (**E**) Lysosomal pH measurements in WT vs. *GRN* KO i³Neurons with linear (left) and 3rd order (right) calibration curve fitting.

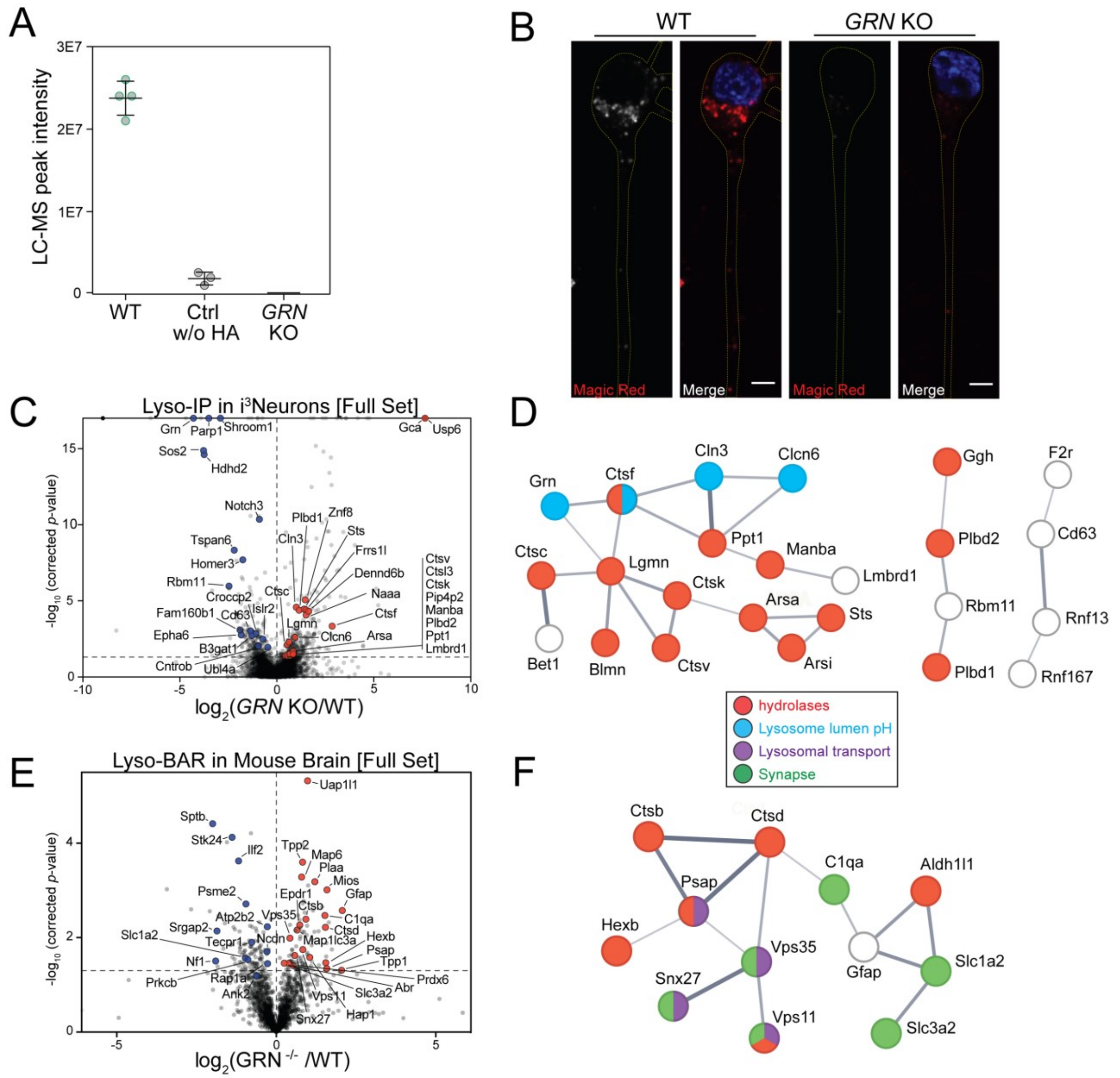

Supplementary Figure S3. Loss of progranulin results in elevated levels of lysosomal catabolic enzymes and decreased cathepsin B activity in human i^{3}Neurons and mouse brains, related to Figure 3. (**A**) PGRN is enrichment in isolated WT lysosomes and is absent in *GRN* KO lysosomes from Lyso-IP proteomics data. (**B**) Additional replicate of Magic Red assay showing reduced cathepsin B activity in *GRN* KO i^{3}Neurons compared to WT. Scale bar is 10 µm. (**C**) Volcano plot of Lyso-IP proteomics in KO vs. WT i^{3}Neurons without filtering, related to Figure 3B. (**D**) Protein network analysis of selected lysosome and synaptic proteins that are significantly changed in *GRN* KO vs. WT Lyso-IP proteomic. (**E**) Volcano plot of Lyso-BAR proteomics in GRN$^{-/-}$ vs. WT mouse brains without filtering, related to Figure 3E. (**F**) Protein network analysis of selected lysosome and synaptic proteins that are significantly changed in *GRN*$^{-/-}$ vs. WT Lyso-BAR proteomics.

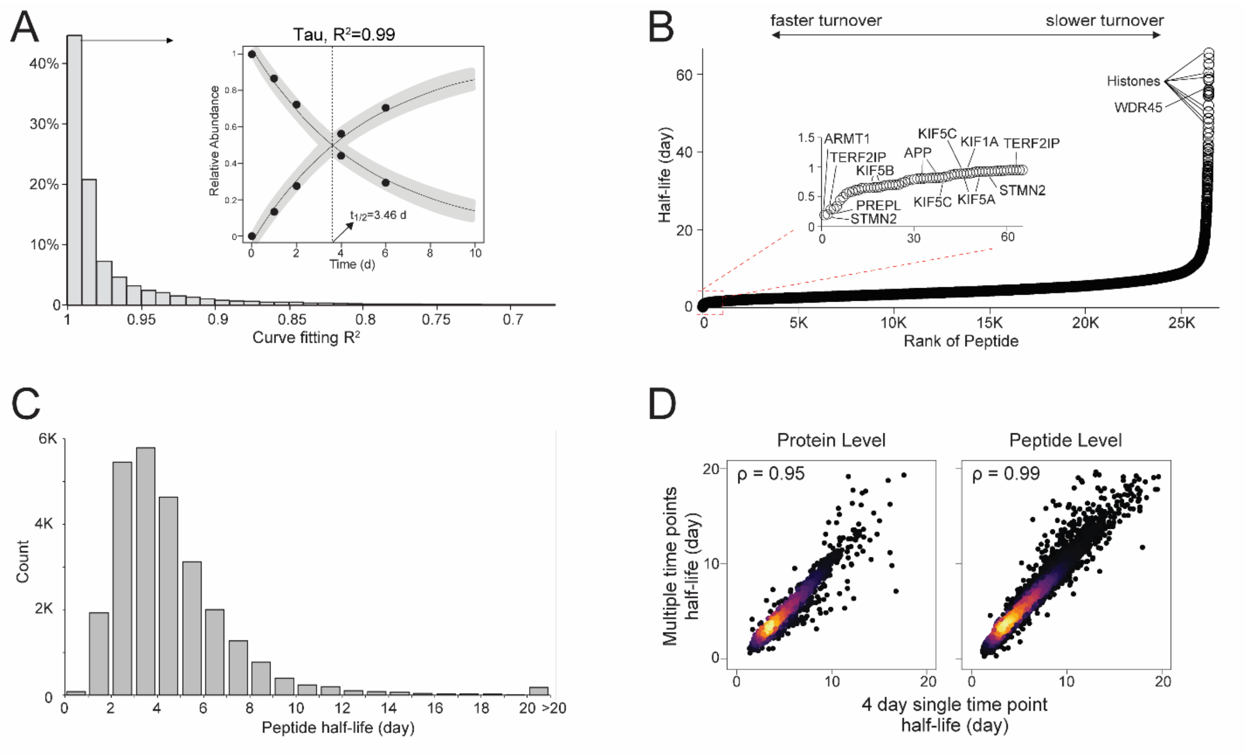

Supplementary Figure S4. Developing dynamic SILAC proteomics in i³Neurons to measure global neuron protein half-lives, related to Figure 4. (**A**) Histogram distribution of peptide level curve fitting R^2 to first-order exponential decay. An example of Tau peptide degradation and synthesis curves is shown in the inset. (**B**) Scatter plot of ranked peptide level half-lives in WT i³Neurons. (**C**) Histogram distribution of peptide half-lives, consistent with protein level results in Figure 4D. (**D**) Scatter plot showing strong correlation of protein/peptide half-lives measured by multiple-time-point method (1, 2, 4, 6 days) and single-time-point method (4 day).

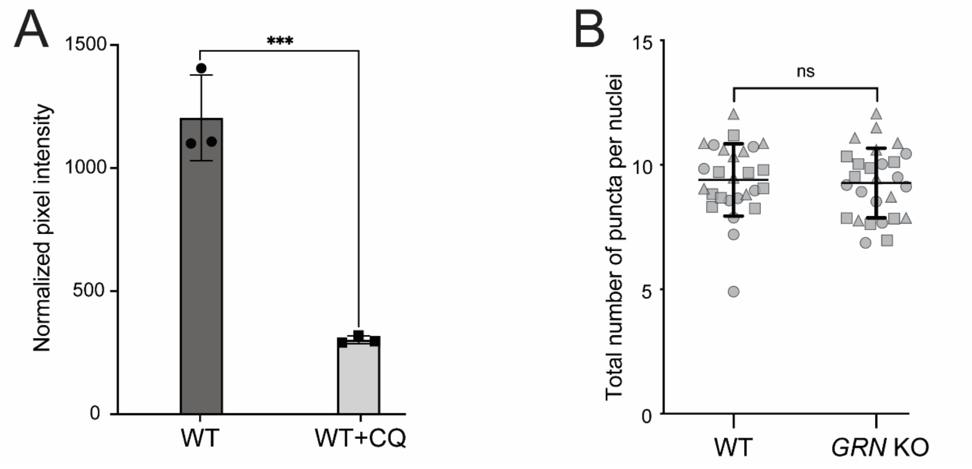

Supplementary Figure S5. DQ-BSA Red Assay to measure lysosomal degradative function, related to Figure 5. (**A**) Chloroquine treatment (30 µM for 12 hours) impairs the lysosomal degradative function with significantly reduced DQ-BSA signals compared to the untreated WT i[3]Neurons (*** denotes *p*-value < 0.001). (**B**) WT and *GRN* KO i[3]Neurons have similar total number of DQ-BSA fluorescent puncta, indicating similar overall levels of endocytosis and lysosomal biogenesis, related to Figure 5I.

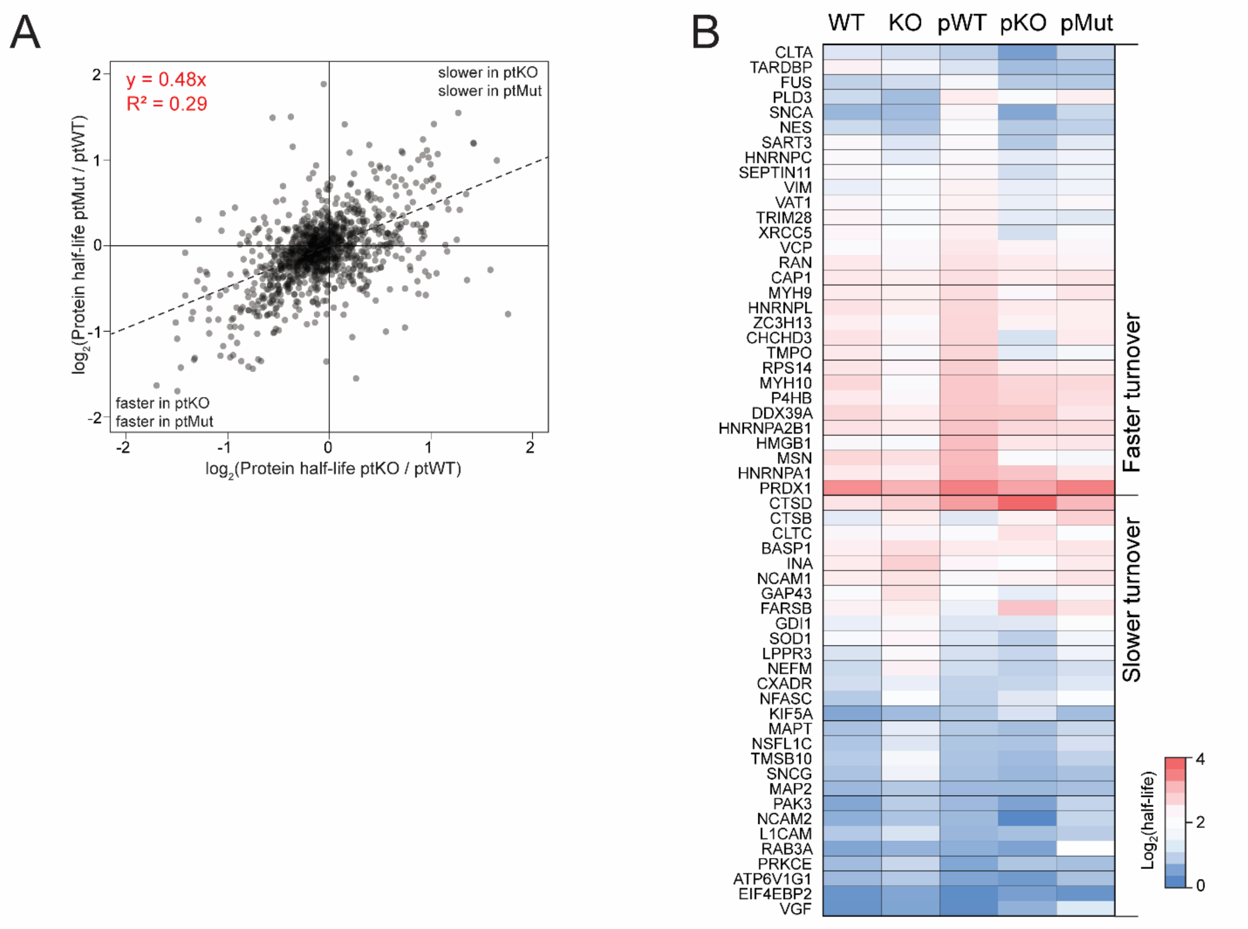

Supplementary Figure S6. **Protein half-life changes caused by progranulin deficiency in GRN-KO, WT, ptKO, ptMut, and ptWT i^{3}Neurons, related to Figure 6.** (**A**) Scatter plot showing potential gene dosage effect of protein half-life changes in ptKO vs. ptWT and ptMutant vs. ptWT i^{3}Neurons. (**B**) Heatmap showing overlapping protein half-lives in WT, *GRN*-KO, ptWT, ptKO, and ptMut i^{3}Neurons. Heatmap colors represent the absolute half-life measurements in days.
